# Species richness drives selection of individuals within wetlands based on traits related to acquisition and utilization of light

**DOI:** 10.1002/ece3.9959

**Published:** 2023-04-07

**Authors:** Lucas Deschamps, Raphaël Proulx, Guillaume Rheault, Nicolas Gross, Christopher Watson, Vincent Maire

**Affiliations:** ^1^ Département des sciences de l'environnement Université du Québec à Trois‐Rivières Trois Rivières Canada; ^2^ Université Clermont Auvergne, INRAE, VetAgro Sup Unité Mixte de Recherche Ecosystème Prairial Clermont‐Ferrand France

**Keywords:** alpha diversity, community ecology, demography, density‐dependent mechanisms, environmental filtering, functional traits, hierarchical models, intraspecific variation

## Abstract

Selection within natural communities has mainly been studied along large abiotic gradients, while the selection of individuals within populations should occur locally in response to biotic filters. To better leverage the role of the latter, we considered the hierarchal nature of environmental selection for the multiple dimensions of the trait space across biological levels, that is, from the species to the community and the ecosystem levels. We replicated a natural species richness gradient where communities included from two to 16 species within four wetlands (bog, fen, meadow, and marsh) contrasting in plant productivity. We sampled functional traits from individuals in each community and used hierarchical distributional modeling in order to analyze the independent variation of the mean and dispersion of functional trait space at ecosystem, community, and species levels. The plant productivity gradient observed between wetlands led to species turnover and selection of traits related to leaf nutrient conservation/acquisition strategy. Within wetlands, plant species richness drove trait variation across both communities and species. Among communities, variation of species richness correlated with the selection of individuals according to their use of vertical space and leaf adaptations to light conditions. Within species, intraspecific light‐related trait variation in response to species richness was associated with stable population density for some species, while others reached low population density in more diverse communities. Within ecosystems, variation in biotic conditions selects individuals along functional dimensions that are independent of those selected across ecosystems. Within‐species variations of light‐related traits are related to demographic responses, linking biotic selection of individuals within communities to eco‐evolutionary dynamics of species.

## Graphical abstract

Within wetlands, some species used intraspecific trait variation to maintain equally dense populations, while others used it to become dominant in favorable conditions.

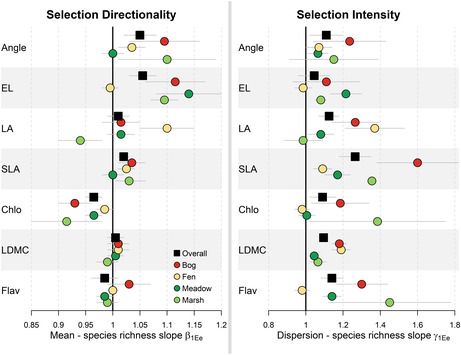

## 1 INTRODUCTION

Selection of individual plants growing within plant communities is often described as a hierarchical sequence of abiotic and biotic filters (HilleRisLambers et al., 2012; Lortie et al., 2004; Weiher & Keddy, 1995). Consequences of these filters on plant communities have been comprehensively explored using functional traits, which are measurable characteristics of individuals linked to their fitness (Violle et al., 2007). When aggregated at the community level, change in trait values is the most widely used tool to reveal how different environments select individuals based on their ability to express traits that allow them to grow and reproduce under a given set of conditions and resources (Shipley et al., 2006). The *directionality* of the selection process describes the displacement of the mean trait value along an environmental gradient (Cavender‐Bares et al., 2004). The *intensity* of the selection measures the amount by which ecological constraints (e.g., stress, disturbance, and biotic interactions) at a given position along the environmental gradient reduce or enlarge the envelope of realized trait values (Laughlin & Joshi, 2015).

**FIGURE 1:**
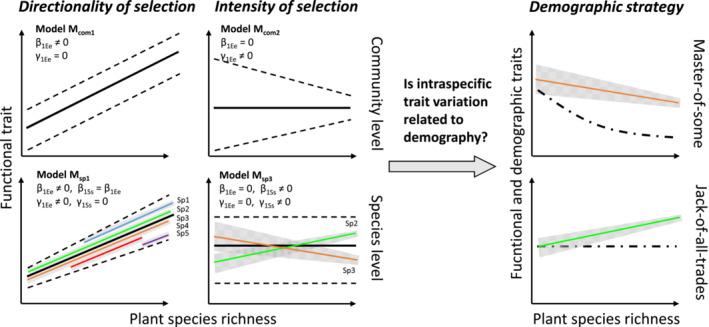
Conceptual framework representing the selection of individuals within communities and the corresponding trait variation along an environmental gradient. Species richness was considered as a biotic gradient, along which intraspecific competition decreases. The selection of individuals by biotic constraints along the gradient can induce a directionality and an intensity of trait variation both at community and species biological organization levels. Using a Bayesian hierarchical model, we disentangled the variation occurring at each level to better understand the selection of individuals within ecosystems. The species richness gradient was repeated within four wetland ecosystems (bog, fen, wet meadow, and fluvial marsh). Directionality was represented by the trait‐richness slope β and the intensity was represented by the trait‐richness parameter γ occurring both at community and species levels. Some of the models are represented in the figure and the full list is available in Table 1.

Selection directionality can be evaluated by calculating the community‐aggregated average of a particular trait, while selection intensity is studied using the statistical dispersion of individual traits within a community (Figure 1, Cornwell & Ackerly, 2009). Variation in community trait mean and dispersion may be due to species turnover, change in species relative abundances or intraspecific adaptations (De Bello et al., 2012). Between ecosystems, relationships between environment and community trait are usually associated with strong species turnover from one environment to another leading to interspecific trait differences and variation in community mean and dispersion (e.g., Bjorkman et al., 2018; Le Bagousse‐Pinguet et al., 2017; Shipley, 2010). Within ecosystems, trait–environment relationships are underexplored, probably because one could consider weak changes in environmental conditions and trait values, which would hamper our ability to detect selection on environmental gradients. However, intraspecific adaptation within ecosystems can lead to changes in trait values that are more important than the ones related to interspecies differences (Messier et al., 2017). As a result, hierarchical analyses of trait variation within and among communities are needed to understand the dominant drivers of selection on environmental gradients (as recently reviewed by Anderegg, 2023).

Within ecosystems, biotic constraints (e.g., intra‐ and interspecific competition, facilitation) often dominate over abiotic constraints and structure the spatial heterogeneity of plant communities (Berdugo et al., 2019; Chalmandrier et al., 2017; Gross, Börger, Duncan, & Hulme, 2013; Gross, Börger, Soriano‐Morales, et al., 2013). Selection directionality in trait–environment relationships may occur among communities when relative fitness advantage of species varies, displacing the community trait mean toward the most competitive strategy in the case of competitive hierarchy (Kunstler et al., 2012), or toward the center of a multimodal distribution when different equivalent strategies coexist (Le Bagousse‐Pinguet et al., 2017). Selection intensity among communities will decrease when the dispersion of trait values is increased by competitive exclusion (Chesson, 2000; Mayfield & Levine, 2010), or by limiting similarity processes that force plants to exploit different strategies to balance inter‐ over intraspecific competition (Grime, 2006; Gross, Börger, Duncan, & Hulme, 2013). To study the response of selection directionality and intensity to the strength of biotic constraints within ecosystems, the local pattern of species richness can be considered as a good candidate (as proposed in Bittebiere et al., 2018). It can strongly vary within a given ecosystem (Huston, 2014; Willis & Whittaker, 2002), and as consequence decreases intraspecific competition and acts as a driver of trait mean and dispersion (Cornwell & Ackerly, 2009; Wolf et al., 2021; Zuppinger‐Dingley et al., 2014).

Within species, selection also operates between individuals (intraspecific trait variations), where both directionality and intensity shape the realized species niche in response to biotic constraints (Austin & Smith, 1989; Levine & HilleRisLambers, 2009). Individuals of different populations may tend to exhibit different mean trait values depending upon the competitive advantage it provides (Hart et al., 2016; Weiner & Thomas, 1986). Trait dispersion of a given species may be reduced when individuals exploit the same specialized resources and limit overlap with neighbors (Hulshof et al., 2013; Violle et al., 2012). On the contrary, trait dispersion expands when neighbors vary in strategies, potentially decreasing competition between conspecifics (Le Bagousse‐Pinguet et al., 2014; Meilhac et al., 2020). Such intraspecific trait variation can be associated with population dynamics using the framework by Richards et al. (2006, Figure 1). *Jack‐of‐all‐trades* strategists are species that exploit their intraspecific variations to maintain their density through contrasting biotic constraints. Individual density of such species will be independent of species richness within the community (Richards et al., 2006). *Master‐of‐some* strategists would instead take advantage of environments with low abiotic constraints to dominate and maintain higher demographic rates. Their individual density will decrease as species richness increases, likely using intraspecific trait variation as a potential way to limit between‐species differences. Rarely, intraspecific trait variation has been associated with changes in demographic descriptors, such as individual density.

Selection directionality and intensity needs to be studied on multiple traits that describe the ability of individual to cope with changes to biotic and abiotic constraints (Cornwell et al., 2006; Spasojevic & Suding, 2012). It has been shown that variation of distinct traits takes place at different levels of biological organization (de Bello et al., 2013; Siefert et al., 2014) and that uncorrelated sets of functional traits rely on different resources and selection processes (Da Silveira Pontes et al., 2015; Maire et al., 2012). To understand environmental selection within ecosystems, functional traits should be selected to represent the independent dimensions along which selection occurs on individuals and percolate up to species and communities.

We designed an original field‐based approach that aims to disentangle selection directionality and intensity at both species and community levels (see Figure 1). We selected four sites corresponding to different wetland ecosystems with a similar climate and characterized by contrasting soil pH and plant productivity. Within each ecosystem, we selected eight natural communities (ca. 20 m^2^ plot) from monodominated to highly diverse communities, establishing a species richness gradient where abiotic differences (including soil pH) are minimized (Rheault et al., 2015). In each community, we randomly sampled individuals and measured key functional traits that are related to nutrient and light acquisition. We formulated hierarchical models with explicit parameters for the mean and dispersion of these functional traits at ecosystem (= site), community (= plot) and species levels. We used this hierarchical experimental design to model trait–environment relationships, with the aim of exploring the following questions:

Q1: How does species richness modify the directionality and intensity of selection within ecosystems, independently of differences between ecosystems?
Q2: Does selection by species richness occur at the species level?
Q3: Does intraspecific selection by species richness relate to *Jack‐of‐all‐trades* and *Master‐of‐some* demographic strategies?

## 2 MATERIALS AND METHODS

### 2.1 Study sites

In the lowlands of the St Lawrence River in Eastern Canada, a species richness gradient was chosen in four highly contrasting natural wetland ecosystems. These wetlands share the same temperate humid climate (mean annual temperature = 5.4°C, mean annual precipitation = 1030 mm, mean growing season length = 112 days) with acidic soils due to the proximity to the granitic Canadian shield. These ecosystems ranked along a soil fertility gradient (soil pH as proxy, Figure S1) and were characterized as bog (Lac‐à‐la‐Tortue, 46°33′15″N 72°39′46″W), fen (Red‐Mill, 46°25′38.9″N 72°29′46.6″W), wet meadow (Sorel Islands, 46°04′12.9″N 73°10′11.1″W), and fluvial marsh (Maskinonge, 46°11′39.1″N 72°59′58.7″W). Within each ecosystem, we selected eight communities of similar area to build a species richness gradient (total number of communities = 31, with only seven communities sampled in the wet meadow). The richness gradient was comparable between ecosystems and ranged from two species to 16 species per similar area (ca. 20 to 25 m^2^, Figure 1a). Importantly, there was no relationship between soil pH and species richness or plant density (Figures S1). The experimental design minimized abiotic differences among communities within ecosystems (Rheault et al., 2015).

### 2.2 Vegetation sampling

Within each community, we sampled 80 individuals (ramets) with at least two mature leaves. We identified species directly in the field and confirmed the identification in the laboratory using the key and description by Brouillet et al. (2002). Sampling was conducted during two campaigns (June 14, 2016–July 5, 2016 and August 22, 2016–September 3, 2016). We used point‐plant distance sampling (Elzinga et al., 1998), conducted simultaneously by two independent harvesters using successive random bearings and distances. At each point, the closest mature plant was harvested, and its distance to the point measured. Plant density within communities were computed using Equation 1, where dens_*p*_ is the density in individuals per m^2^ of community *c*, *n* is the number of individuals harvested within the community, and *d*_*ci*_ is the distance, in cm, between the point and the individual *i* of community *c*:

(1) $$\mathrm{den}s_{c}=\frac{1}{10^{4}}{\frac{\sum_{i=1}^{n}d_{\mathrm{ci}}}{n}}^{2}$$

### 2.3 Trait measurements

We selected a set of plant functional traits that are linked to different biological functions (Table S1). The area (LA), extended length (EL), and angle of the leaves are linked to vertical space occupation strategies that are used to compete for light interception (Hikosaka & Hirose, 1997; Weiner & Thomas, 1986), while leaf chlorophyll content and specific leaf area (SLA) represent the fine‐scale adaptation to optimize light utilization (Kull & Niinemets, 1998; Poorter et al., 2012). Leaf dry matter content (LDMC) and flavonoid content are related to nutrient conservation and involved in response to stress and/or herbivory (Hodgson et al., 2011; Izaguirre et al., 2007). On each individual, we measured the length from the ground to the edge of deployed leaves (EL, cm). We collected the last mature leaf, photographed it, and weighted fresh and dry leaf mass to calculate LA, LDMC, and SLA following the protocols described by Pérez‐Harguindeguy et al. (2013). Chlorophyll content is the concentration of chlorophyll in the leaf epidermis (μg cm^−2^), and flavonoid content is an index of flavonoids concentration in this superficial layer, which is related to phenol accumulation. We measured the latter two variables using a portable Dualex instrument (Force‐A, Orsay, France), which uses a combination of fluorescence signals at various excitation bands to quantify pigments and chemical compounds. This method that we validated for our species pool (Figure S2), has been successfully used to study leaf phenology (Mattila et al., 2018), and the response of leaf metabolism to both nutrient (Scogings, 2018) and light manipulation (Agati et al., 2011).

### 2.4 Data analysis

#### 2.4.1 Modeling framework

We used a Bayesian distributional modeling framework as proposed by Rigby and Stasinopoulos (2005) to model trait and plant density distributions at ecosystem, community, and species levels (see sAppendix S1 for full details on the Bayesian modeling). We used two distribution probability families to describe trait variation between individuals. For traits with only positive values (except LDMC), we used a Gamma distribution, whereas we modeled LDMC using a Beta distribution. We parameterized the Gamma distribution using the mean (*μ*) and dispersion (*σ*) parameters while we used a precision parameter (*ϕ*) to quantify the dispersion of the Beta distribution. We used the parameters of trait distribution as proxies of the selection directionality (mean *μ*) and the selection intensity (dispersion *σ* or precision *ϕ*).

#### 2.4.2 Selection by plant species richness within ecosystems (Q1)

We used all available trait data (raw data = 2480) to focus on the unique response of traits to plant species richness gradient within ecosystems. The most complex model (*M*_com3_) describing the distribution from which the trait value of the *i*th individual, *y*_*i*_, is drawn, was written as follows:

(2) $$y_{i}∼f\left(\mu_{i},\sigma_{i}\right)$$

(3) $$g_{1}\left(\mu_{i}\right)=\beta_{0}+\beta_{T2}+\beta_{\mathrm{Ee}}+\beta_{\mathrm{Cc}}+\beta_{1\mathrm{Ee}}D_{c}$$

(4) $$g_{2}\left(\sigma_{i}\right)=\gamma_{0}+\gamma_{T2}+\gamma_{\mathrm{Ee}}+\gamma_{\mathrm{Cc}}+\gamma_{1\mathrm{Ee}}D_{c}$$

*where f()* is a probability distribution parameterized in term of *μ* and *σ* (*or ϕ*), while *g*_1_ *()* and *g*_2_ *()* are link functions. *β*_0_ and *γ*_0_ are intercepts for the first sampling campaign, while *β*_*T*2_ and *γ*_*T*2_ are the deviations for the second campaign for the mean and the dispersion of the distribution, respectively. *β*_*Ee*_ and *β*_*Cc*_ are deviation parameters describing how the mean of each ecosystem *e* and community *c* differ from the overall mean of each campaign *T*. *γ*_*Ee*_ and *γ*_*Cc*_ describes differences in dispersion between ecosystem and community, respectivelyy. *β*_*Cc*_ and *γ*_*Cc*_ are treated as hierarchical parameters, normally distributed with estimated variances. *β*_1*Ee*_ and *γ*_1*Ee*_ are the ecosystem‐specific slopes describing the effect of an increase in one species on the mean and dispersion of community trait distribution, respectively. *D*_*c*_ is the species richness of community *c*.

To respond to Question 1, we compared four candidate models and we evaluated their predictive ability to fit observed data. The reference model, *M*_com0_, described trait distributions of each community as a series of intercepts. *M*_com1_ included a slope per ecosystem describing the link between species richness and the mean of the community trait distribution, while *M*_com2_ included a slope linking species richness to trait dispersion. *M*_com3_ included both slopes, assuming that biotic selection exhibited both directionality and intensity. If the predictive ability of *M*_com3_ model was better than previous models, it would mean that both directionality and intensity were important to describe trait distribution at community level. To test the importance of intraspecific variation to respond to the richness gradient and initiate the response to Q2, we refitted *M*_com3_ by replacing observed individual values by mean species value (*M*_com3’_), estimated from a model containing an intercept for each campaign and a hierarchical intercept per species, distributed normally with estimated standard deviation. By removing intraspecific trait variation, we tested whether the response of community trait distributions were only due to variation in species abundance.

#### 2.4.3 Selection by plant species richness within species (Q2)

We explored the role of intraspecific variation to respond to plant species richness by using a subset of species, which occurred within a minimum of four communities and at both extremities of the species richness gradient of their ecosystem. Eleven species were selected across ecosystems (*n* = 1045). The most complex hierarchical model determined both mean and dispersion as a function of intercepts and species richness, with equations for *μ* and *σ* (*or ϕ*):

(5) $$y_{i}∼f\left(\mu_{i},\sigma_{i}\right)$$

(6) $$g_{1}\left(\mu_{i}\right)=\beta_{0}+\beta_{T2}+\beta_{\mathrm{Ee}}+\beta_{\mathrm{Cc}}+\beta_{\mathrm{Ss}}+\beta_{1\mathrm{Ss}}D_{c}$$

(7) $$g_{2}\left(\sigma_{i}\right)=\gamma_{0}+\gamma_{T2}+\gamma_{\mathrm{Ee}}+\gamma_{\mathrm{Cc}}+\gamma_{\mathrm{Ss}}+\gamma_{1\mathrm{Ss}}D_{c}$$

with *β*_*Ss*_ and *γ*_*Ss*_ being species‐specific deviation parameters for mean and dispersion, and *β*_1*Ss*_ and *γ*_1*Ss*_ species‐specific slopes between species richness and mean and dispersion, respectively. They are all hierarchical parameters distributed multinormally with an estimated covariance matrix. The reference model, *M*_sp0_, contained a slope per ecosystem linking both species mean and dispersion to species richness. *M*_sp1_ included a slope per ecosystem for dispersion, but a slope per species linking species richness to species mean trait values, while *M*_sp2_ included the opposite syndrome. *M*_sp3_ allowed mean and dispersion of each species to move idiosyncratically with the number of species with which they grow. If the predictive ability of M_sp3_ model was better than previous models, it would mean that both directionality and intensity were important to describe trait distribution at the species level.

#### 2.4.4 Jack‐of‐all‐trades/Master‐of‐some strategies (Q3)

To explore which species exhibit *Jack‐of‐all‐trades* and *Master‐of‐some* strategies in response to species richness (Figure 1), we summarized for each species in a given community its intraspecific variation in traits and density, consistent with the framework presented by Richards et al. (2006). Given the importance of vegetative reproduction in wetlands (7.9% of our individuals were harvested with flower or fruits and most species have underground vegetative buds), we considered density (individuals per m^2^) as a good proxy of species demographic performance. As such, a population with high density will have lower risk of mortality and greater capacity for reproduction, compared with a low‐density population (Santamaria et al., 2003).

To explore responses of species density along the plant species richness gradient, we used a comparable model of *M*_sp3_, with *y*_i_ being species density. We interpreted the species strategy as *Jack‐of‐all‐trades*, whenever mean slope of the density model did not show substantial variation. Then, we explored whether trait variation showed consistency, either for mean and dispersion or for trait identity. In contrast, the *Master‐of‐some* strategy represented species that substantially increased their density in low diversity communities, with or without detected trait variation. Then, we explored whether each strategy displayed substantial covariations between trait slopes (for mean and dispersion) and density slope for mean at species level.

#### 2.4.5 Bayesian modeling details

The Bayesian distributional modeling framework allows modeling each parameter of a given probability trait distribution with an independent equation (see Appendix S1). As such, we quantified the independent role of the mean and dispersion on trait variation at ecosystem, community, and species levels (Figures S4–S19). It is not appropriate to use a generalized linear model framework for our purpose as it relies on a fixed dispersion assumption (Cepeda‐Cuervo, 2015; Smyth, 1989). We considered that parameters show substantial response to species richness whenever the 90% credible interval of their standardized estimate did not include 1. Posterior distributions of parameters have been sampled by four independent chains using the *No‐U‐Turn Sampler* implemented in the *R* package *brms* (Bürkner, 2017). We visually examined all chains, posterior distributions, and posterior‐predictive checks to ensure the model accuracy, to avoid divergent iterations and to ensure chain convergence (Figures S20–S47). Model performance was evaluated by the mean of weights based on the stacking of predictive distribution, with a model providing better predictions of future data having a higher weight. We estimated model weights to maximize leave‐one‐out predictive density of a complete model containing all submodels. This method is the least sensitive to overfitting in Bayesian modeling, and includes uncertainty about every model during weights estimation (Yao et al., 2017).

## 3 RESULTS

### 3.1 Community trait distribution response between ecosystems

**FIGURE 2:**
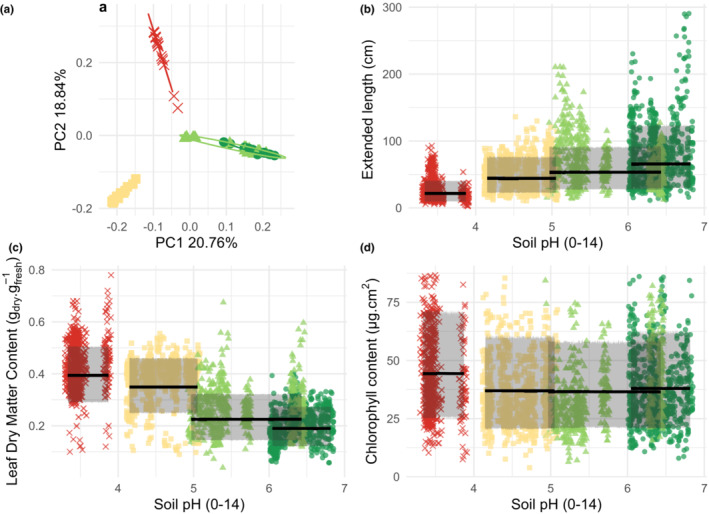
Differences between ecosystems in term of species and functional traits. Panel (a) represents a principal component analysis of Hellinger transformed species density. Lines represents 90% ellipses capturing 90% of the points. Panels (b–d) represent observed trait values with predictions of the model containing an intercept per ecosystem for both mean and dispersion. Lines represent mean predicted value. Shaded areas represent 90% predictive interval of community trait distribution. Panels (b, c) represent mean Leaf Dry Matter Content (LDMC) and both Leaf Area (LA) mean and dispersion varying monotonically between ecosystems. Panel (d) represents an absence of between‐ecosystem monotonic variation for chlorophyll content per unit area of leaves. *Red cross* = bog, *yellow square* = fen, *light green triangles* = wet meadow, *dark green dots* = fluvial marsh.

We observed strong functional differences and turnover of species between each wetland (Figure 2). Mean and dispersion of traits (*β*_*Ee*_ and *γ*_*Ee*_, respectively, in Equation 1) related to space filling and light acquisition (EL and LA) increased monotonically along the soil pH gradient, while the mean of traits related to nutrient conservation (LDMC and flavonoids) decreased (Figure 2 and Figures S6, S7, S11, and S12). Leaf angle, SLA, and chlorophyll content varied between ecosystems, but did not show a monotonic ordered pattern in mean and dispersion along the soil pH gradient (Figures S8–S10).

### 3.2 Community trait distribution response within ecosystems

**FIGURE 3:**
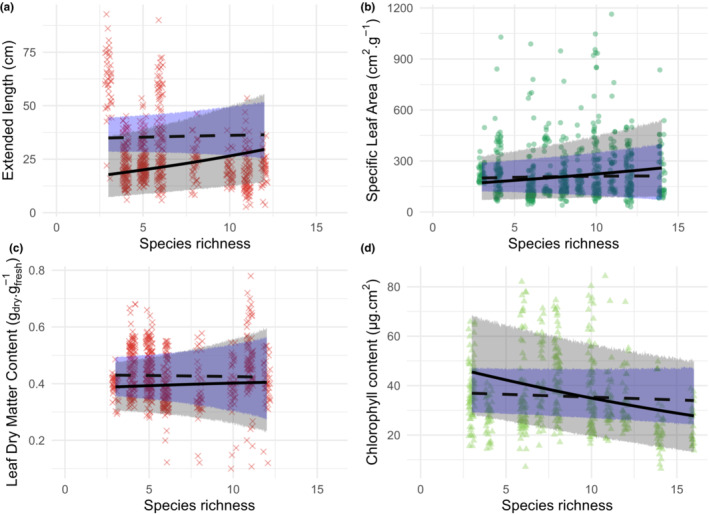
Examples of response of community trait distribution to within‐ecosystem species richness gradient. Panels a‐d represents the response of Extended Length, Specific Leaf Area, Leaf Dry matter Content and Leaf Chlorophyll Content to species richness, respectively. Panels a and c represent examples of selection directionality and intensity in bog wetlands, respectively, while panels b and d represent examples of positive and negative selection directionality in marsh and meadow wetlands, respectively. Solid lines represents mean predicted values based on observed data (considering intraspecific trait variation, ITV, *M*_com3_ in Table 1), while dashed lines represents mean predicted values of models fitted using species mean values (without ITV *M*_com3’_ in Table 1). Gray and blue shaded area represents 90% predictive interval of community trait distribution with and without ITV, respectively. *Red cross* = bog, *light green triangles* = wet meadow, *dark green dots* = fluvial marsh.

Local gradient of species richness observed within ecosystem strongly shaped the functional trait distribution of local communities. Including the species richness of the community (and its associated parameters, *β*_1*Ee*_ and *γ*_1*Ee*_ in Equations (2), (3), (4)) improved the fit of the models for all traits (Table 1). *M*_com0_ was always the least supported model. For all traits except chlorophyll, *M*_com3_ was always better supported than *M*_com1_ and *M*_com2_, which indicated that the inclusion of both mean and dispersion parameters improved the fit of the models (Table 1). Finally, for all traits except LDMC, exclusion of the intraspecific variation in trait values reduced the fit of the models (Table 1). Without intraspecific variation, the slopes between species richness and trait mean or dispersion were consistently underestimated (Figure 3), which indicated the need to include trait selection operating at the species level. Together, these results indicate that trait–environment relationships within ecosystems are better predicted by considering (i) local variation in species richness, (ii) the selection directionality and dispersion, and (iii) the observed intraspecific variation.

**TABLE 1:** Stacking weights of competing models describing the relationships between trait variation (directionality and intensity) and species richness at community and species levels. Stacking weights varies from 0 to 1, 0 indicating null probability that the model can represent trait variation and 1 indicating a complete representability.

Model	Param.	Selection	Angle	EL	LA	SLA	Chlo	LDMC	Flav
Community level									
*M*_com0_	Int.	None	0.45	0.01	0.09	0.03	0.05	0	0.03
*M*_com1_	*β*_1*E*_	Directionality	0	0	0.20	0	**0.84**	0	0
*M*_com2_	*γ*_1*E*_	Intensity	0.09	0.01	0	0.13	0	0.17	0.07
*M*_com3_	*β*_1*E*_, *γ*_1*E*_	Both	**0.47**	**0.98**	**0.71**	**0.83**	0.11	**0.79**	**0.86**
*M*_com3’_	no ITV	Both, no ITV	0	0	0	0	0	0.04	0.04
Species level									
*M*_sp0_	*β*_1*E*_, *γ*_1*E*_	None	**0.60**	0.32	0.23	0.30	0.31	**0.80**	**0.55**
*M*_sp1_	*β*_1*S*_, *γ*_1*E*_	Directionality	0	0	0	0	**0.69**	0	0
*M*_sp2_	*β*_1*E*_, *γ*_1*S*_	Intensity	0	0	0	0.08	0	0	0
*M*_sp3_	*β*_1*S*_, *γ*_1*S*_	Both	0.36	**0.68**	**0.77**	**0.62**	0	0.20	0.45

*Note*: *Community level* (*M*_com_ *models*): *M*_com0_ contained only intercepts for campaign, ecosystem, and community (the last two considered as a hierarchical parameter with estimated variance). *M*_com1_ and *M*_com2_ estimated a slope per ecosystem between species richness and mean and dispersion of trait distribution, respectively. *M*_com3_ estimated the ecosystem‐specific slopes for both mean and dispersion, considering intraspecific trait variation (ITV) between individuals and campaign. *M*_com3’_ was based on *M*_com3_ but did not consider ITV. *Species level* (*M*_sp_ models): *M*_sp0_ is a model containing categorical effects and a slope per ecosystem linking species richness to traits mean and richness of each frequent species within each ecosystem. In *M*_sp1,_ trait mean is modeled with a slope per species and dispersion per ecosystem, while *M*_sp2_ contained the opposite syndrome. Finally, *M*_sp3_ contains slopes per species for both mean and dispersion. Parameters *β* and *ɣ* represent slopes between species richness and mean and dispersion of trait distribution, respectively. Indices *E* and *S* mean that the slopes were estimated for each ecosystem or for each species, respectively. For each trait and at each biological organisation level, bold value indicates the best competing model to predict trait variation.

Abbreviations: Angle, leaf angle; Chlo, leaf chlorophyll content; EL, extended length; Flav, leaf flavonoid content; LA, leaf area; LDMC, leaf dry matter content; SLA, specific leaf area.

**FIGURE 4:**
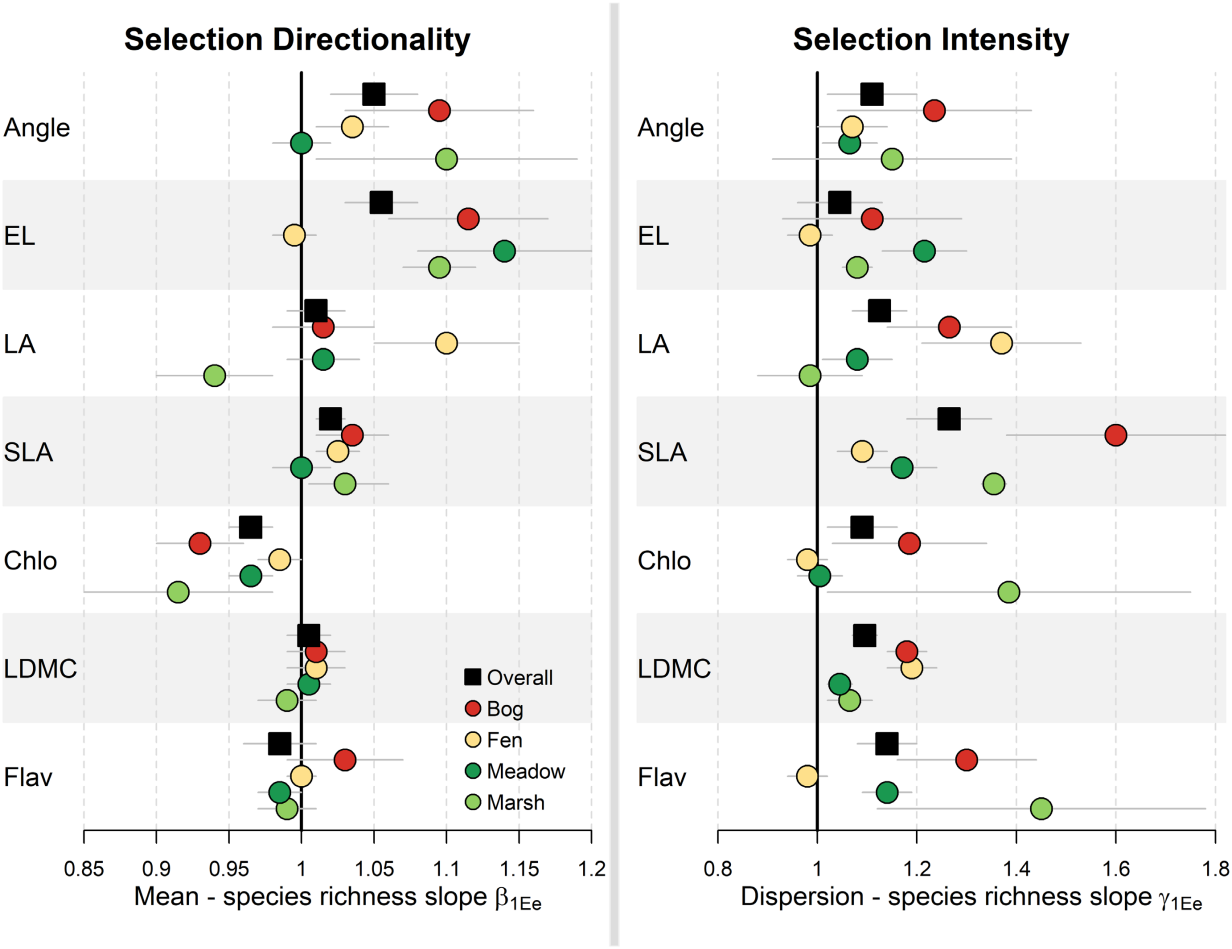
Summary of estimated community trait distribution in response to plant species richness (*M*_com3_ model in Table 1). Directionality represents the mean of the distribution (*μ* in Equation 2), while intensity represents its dispersion (*σ* for gamma distribution in Equation 2) or its precision (*ϕ* for the particular beta distribution of LDMC in Equation 2). We displayed 90% credible intervals of exponentiated slopes (parameter *β*_1*E*_ for *μ* and parameter *γ*_1*E*_ for *σ* and *ϕ* in Equations (2), (3), (4)). The value is interpreted as a multiplicative term of trait mean or dispersion when the community include one more species. Negative directionality between species diversity and a given trait is characterized by mean < 1, whereas mean > 1 characterized positive directionality. Higher selection intensity in response to species richness is characterized by dispersion value <1, whereas lower selection intensity by values >1. When interval includes 1, no trend of directionality or intensity is detected. The precision parameter *ϕ* for the particular beta distribution of LDMC was modified to be interpreted likewise the parameter *σ* for gamma distribution. Overall represents the global mean response across the data set. Predictions are available in Table S2 and Figures S5–S11. Angle, leaf angle; Chlo, leaf chlorophyll content; EL, extended length; Flav, leaf flavonoid content; LA, leaf area; LDMC, leaf dry matter content; SLA, specific leaf area.

The effect of plant species richness was trait‐specific (Figures 3, 4 and Figures S6–S12). The mean of traits related to vertical space (Angle and EL) and fine‐scale light utilization (SLA and chlorophyll) responded to species richness, suggesting a directional selection toward particular values, whereas no substantial trends were detected for LA, LDMC, and flavonoid content. More diverse communities were dominated by individuals with more erect, longer, and thinner leaves but with lower chlorophyll content than individuals in less diverse communities. This result held in all ecosystems, except for wet meadows, where only EL and chlorophyll traits followed the same patterns. We did not observe directional selection of traits related to resource conservation strategy within ecosystems (LDMC and flavonoids), which differed from the selection that occurred between ecosystems on these particular traits. We also showed that species richness led to higher dispersion of trait values for all traits including those related to vertical space filling, fine‐scale light utilization, and resource conservation (Figures 3, 4 and Figures S6–S12). More specifically, communities that are more diverse exhibited greater trait variation among individuals.

### 3.3 Species trait distribution response within ecosystems (Q3)

**FIGURE 5:**
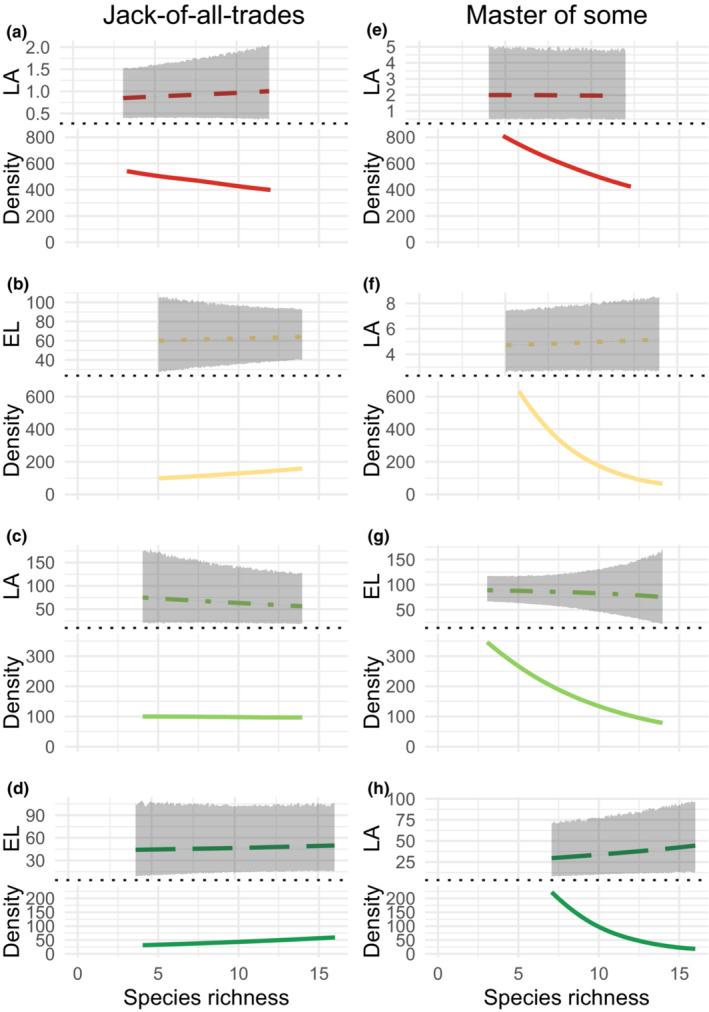
Model predictions of species morphological trait distribution (*M*_sp3_ model in Table 1) and demography (relative density) in response to species richness within communities. Predictions are organized in two columns according to the two demographic strategies that species deployed within ecosystems. The *Jack‐of‐all‐trades* strategy represented species with stable relative density, but with substantial trait variation, represented by displacement of the mean and/or increase/decrease in trait distribution. The *Master‐of‐some* strategy represented species that substantially increased their relative abundance in low diverse communities, reaching dominance. Trait variation is not mandatory for detecting this strategy. Full line represents the species median trait value, while the gray area represents the 90% predictive interval of trait distribution. Dashed line represents the median predicted relative density of each species. LA, Leaf area (cm^2^); EL, Extended length (cm); Density, Relative density (ind m^−2^). Species are *Chamaedaphne calyculata*, *Eriophorum virginicum*, *Carex lasiocarpa*, *Carex sec. phacocystis*, *Typha latifolia*, *Acorus calamus*, and *Lythrum salicaria*.

Within species, selection occurred along the gradient of plant species richness with a response of both the mean and the dispersion for EL, LA, and SLA (*β*_1*S*_, *γ*_1*S*_ in Table 1, Species level) and the mean for chlorophyll, whereas mean and dispersion of other traits did not respond. Table 2 and Figure 5 show that species responses of trait distribution to species richness were idiosyncratic, that is, mean and dispersion of EL, LA, and SLA either substantially increased or decreased according to species (see Figures S13–S19 for details).

**TABLE 2:** Summary of estimated species slopes in the relationships of plant functional and demographic traits with plant species richness.

Ecosyst.	Species	Para.	Angle	EL	LA	SLA	Chlo	LDMC	Flav	Density	Strategy
Bog	*Chamaedaphne calyculata*	μ	0	0	**+**	0	0	0	0	Stable	Jack
		σ/*ϕ*	0	**+**	**+**	0	0	0	0		
	*Eriophorum virginicum*	μ	0	0	0	**‐**	0	0	0	Decrease	Master
		σ/*ϕ*	0	0	0	**+**	0	0	0		
Fen	*Carex lasiocarpa*	μ	0	0	0	0	0	0	0	Stable	Jack
		σ/*ϕ*	0	**‐**	0	**‐**	0	0	0		
	*Carex oligosperma*	μ	0	0	0	0	0	0	0	Decrease	Master
		σ/*ϕ*	**+**	0	**+**	**+**	**‐**	**‐**	0		
	*Carex sect. Phacocystis*	μ	0	0	**‐**	0	0	0	0	Decrease	Master
		σ/*ϕ*	**+**	0	0	**+**	**‐**	**‐**	0		
Meadow	*Acorus calamus*	μ	0	0	**+**	0	0	0	0	Decrease	Master
		σ/*ϕ*	0	**+**	0	0	0	0	0		
	*Lythrum salicaria*	μ	0	0	0	0	0	0	0	Stable	Jack
		σ/*ϕ*	0	0	0	0	0	0	0		
Marsh	*Acorus calamus*	μ	0	0	0	0	0	0	0	Decrease	Master
		σ/*ϕ*	**+**	**+**	0	0	0	0	0		
	*Comarum palustre*	μ	0	0	**+**	0	0	0	0	Stable	Jack
		σ/*ϕ*	0	0	0	0	0	0	0		
	*Lythrum salicaria*	μ	0	0	**‐**	0	0	0	0	Stable	Jack
		σ/*ϕ*	0	**+**	**+**	0	0	0	0		
	*Thypha latifolia*	μ	0	0	**‐**	0	0	0	0	Stable	Jack
		σ/*ϕ*	0	0	**‐**	0	0	0	0		

*Note*: Parameters were recovered from the best model for each trait, presented in Table 1 for species level (parameter *β*_1*E*_ for *μ* and parameter *γ*_1*E*_ for *σ* and *ϕ* in Equations (5), (6), (7)). For sake of clarity, we resumed slopes by symbols representing their sign (see Table S3 for details and Figures S12–S18 for visual representation). We considered the inclusion of one in the 90% credible interval of parameters to attribute a sign, otherwise zero is represented. For each trait, symbols blue ‘+’ and red ‘–’ represented substantial positive and negative, respectively, variation in mean trait value (*μ*) and dispersion (*σ*, *ϕ*) in response to variation in plant species richness within the community. For the density, “decrease” represented substantial variation of species mean relative density with species richness, while “stable” indicated no substantial variation. A species is considered as a *Master‐of‐some* strategist whenever its density decreases with or without presence of intraspecific trait variation in response to plant species richness. In contrast, a species is considered as a *Jack‐of‐all‐trades* strategist, whenever it maintains its density and shows intraspecific trait variation in response to species richness. See abbreviations in Table 1.

**FIGURE 6:**
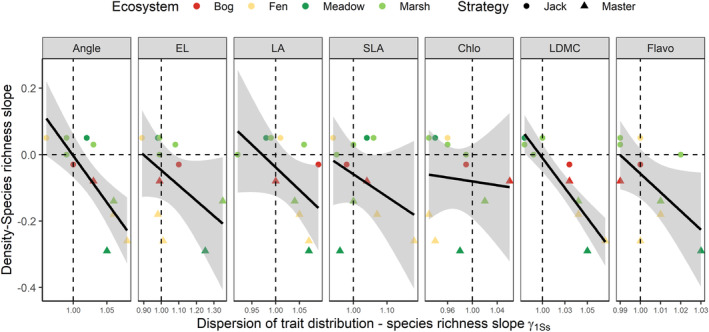
Relationship between the slope of the density/species richness gradient and the dispersion of species trait distribution. Both variables are presented in Table 2. Each point represents a species, which is colored according to the ecosystem and shaped according to the two demographic strategies, *Jack‐of‐all‐trades* and *Master‐of‐some*. The strategy is determined in Table 2. Fitting lines represent mean predicted value. Shaded areas represent 90% predictive interval. Zero horizontal line represent the absence of relationship between species richness and density. One vertical line represents the absence of trait dispersion when species richness increases of one within the community. Higher selection intensity in response to species richness is characterized by dispersion value <1, whereas lower selection intensity by values >1.

We detected two contrasting species‐level responses to the plant species richness gradient. The *Jack‐of‐all‐trades* strategy was found in species with fixed or slightly increasing median relative density along the richness gradient, but with contrasting trait distributions (Figure 5 and Table 2). This occurred both on mean and on dispersion of traits related to light and space acquisition, with the best out‐of‐sample predictions of EL, LA, and SLA provided by the model with species‐specific slopes within each ecosystem (Table 1). For example, *Typha latifolia* exhibited lower and less variable LA in richer fluvial marsh communities, with a median leaf area of 73.9 cm^2^ (±45.4, Table S1) in a less diverse community and 56.8 (±30.1) cm^2^ when growing in a more diverse community (Figure 5). However, in the wet meadow, *Lythrum salicaria* trait mean and dispersion, as well as density, did not change along the richness gradient (Table 2 and Figure 5). Conversely, *Master‐of‐some* strategists were detected when species were highly dense in less diverse communities, but were less dense in more diverse communities. For example, in the fen, there was on average 640 (±70) *Carex oligosperma* individuals per m^2^ in poor communities, and 66 (±4.4) individuals in highly diverse communities. Species showing such a density response tended to present more dispersed distributions of leaf angle (probability of 0.60 ± 0.06) but less dispersed distributions of LDMC (probability of 0.70 ± 0.06) (Figure 6).

Figure 6 shows that the ability of species to maintain the population density along the plant species richness gradient was related to the dispersion of species trait distribution, whereas there was no substantial covariation with the mean (data not shown). As the dispersion of traits increased within the population, the stronger the negative relationship between population density and plant species richness (Pearson correlation coefficient *ρ* = −0.69 for Angle and *ρ* = −0.71 for LDMC).

## 4 DISCUSSION

We show *in‐natura* that within‐ecosystem trait variations can be strongly and consistently structured by species richness and biotic interactions. We detected differences in the distribution of functional traits along a gradient of plant species richness in four contrasting wetlands. Our study reveals that multiple filters are detectable at different levels of biological organization and are acting concomitantly to shape the distribution of traits along environmental gradients. This supports recent studies showing that the drivers of trait variations may differ within and between species (e.g., Anderegg et al., 2018), or while sampling at different spatial scales (Messier et al., 2017).

### 4.1 Species turnover drove selection to soil fertility between ecosystems

Between ecosystems, selection following a complete species turnover occurred on both the directionality and intensity of the distribution of traits related to space occupation (EL and LA) and to nutrient conservation (LDMC and flavonoid content). The increase in space occupation and decrease in nutrient conservation characterized the directionality from less to more fertile ecosystems, respectively. Selection intensity showed an opposite pattern, trait dispersion of EL, LA, and flavonoid increasing along the fertility gradient. Both selection directionality and intensity on these traits are consistent with known functional trade‐offs between nutrient conservation and growth along fertility gradients (Jager et al., 2015), where plants selected in the more fertile ecosystems are those with the lowest investment in leaf longevity but with the greatest ability to compete for space (Grime, 1977; Wright et al., 2004).

While an almost complete turnover of species drove differences between ecosystems, SLA and fine‐scale light acquisition traits (chlorophyll content and leaf angle) did not show clear patterns of selection directionality and intensity along the pH gradient. Using a hierarchical modeling structure, we were able to show that these traits nevertheless responded to the gradient of plant species richness within ecosystems.

### 4.2 Within ecosystems, species richness modified the directionality and intensity of selection

Within wetlands, the species richness gradient selected individuals from a common species pool, by changing both the directionality and the intensity of the trait distribution (Figures 3 and 4). For all traits, selection intensity decreased with increasing plant species richness, revealing a divergence of individuals toward more heterogeneous leaves when communities are more diverse. This characteristic has likewise been described in temperate grasslands (Le Bagousse‐Pinguet et al., 2014, 2015).

With increasing species richness, individuals were, on average, more erect, taller and exhibited lower chlorophyll‐investment in leaves. Such responses likely result from the well‐known trade‐off in resource allocation to adapt to light conditions: Gommers et al. (2013) contrast species using strategy of shade tolerance (increasing light capture with high SLA but low chlorophyll) to species using a strategy of shade avoidance (increasing light use with high chlorophyll and low SLA). Following this shade tolerance/avoidance framework, shade tolerance strategy would be selected within communities that are more diverse. We also observed that those communities are particularly more erect and taller, a hyponastic syndrome that occurs to adapt to lower light conditions (Millenaar et al., 2009). Whereas selection for light structures plant communities in nutrient‐rich environments (e.g., Maire et al., 2012), this process has rarely been highlighted in resource‐poor environments (but see Wiktor & van Diggelen, 2004). Selection directionality and intensity of space‐ and light‐related traits was the primary consequence of response to species richness gradient.

We also show that more diverse communities had leaves with higher SLA and more dispersed values in SLA, LDMC, and flavonoids than in less diverse ones. This reveals that species richness selects for communities with higher resource acquisition rate and more diverse resource acquisition strategy (Wright et al., 2004). These responses were observed in diversity experiments in grassland and forest ecosystems (Davrinche & Haider, 2021; Siebenkäs et al., 2017) and was interpreted as a limiting similarity process enhancing complementarity in light and nutrient resources (Gubsch et al., 2011; McKane et al., 2002). On the opposite, in communities that were less diverse, dominant individuals converge toward graminoid‐like species with high nutrient conservation (e.g., *Typha* in nutrient‐rich ecosystem, *Eriophorum* and *Carex* in nutrient‐poor ecosystem). While responding mainly to the abiotic gradient across ecosystems, LDMC and flavonoid also showed a biotic selection, which was more intense in less diverse communities, and particularly in acidic ecosystems. While the statistical dispersion of LDMC and flavonoid values increased with species richness in more acidic ecosystems, the mean did not change. This suggests that convergence toward conservative strategy in less diverse communities operated through competitive exclusion or limiting‐similarity processes rather than through competitive hierarchy, which is more associated with directional selection. Of special note, it would have not been possible to detect this within‐ecosystem pattern on LDMC and flavonoid without a statistical model including the hierarchy between ecosystem and community levels and considering both the mean and the dispersion of trait distributions.

### 4.3 Within species, intraspecific trait variation respond to plant species richness

Within species, functional responses to the species richness gradient were observed through the adjustment of both the mean and the dispersion of trait values. As such, selection directionality and, particularly, intensity occurred also within species, but on a different dimension of plant functional strategy. Within species, selection intensity was particularly associated with EL, LA, and SLA. This suggests that the species richness gradient selected for species able to use intraspecific trait variation to adapt to changes in space and/or light resources. Importantly, we found that this adaptation at the species level were idiosyncratic, that is, it varied between species. For example, *Lythrum salicaria* decreased in leaf area with increasing species richness, while LA increased for *Comarum palustre*. Idiosyncratic responses at species level occur usually along environmental gradients (e.g., along altitudinal gradient in Umaña & Swenson, 2019) but is not necessarily the rule as consistent behaviors can also be found (e.g., along aridity gradient in Dong et al., 2020). These results suggest that species adjusted their space and light acquisition trait in multiplying ways rather using a unique response to species richness gradient. Overall, our study highlights species richness as a strong structuring driver of trait variation both at community and at species levels with different species exhibiting contrasting strategies to adapt to their neighbors.

### 4.4 Species showed Jack‐of‐all‐trades and Master‐of‐some strategies

Within species, we considered intraspecific trait variation in response to plant species richness in regard with the demographic strategies established by Richards et al. (2006). *Jack‐of‐all‐trades* species are able to maintain high demographic rates across a gradient of environmental conditions. Our result demonstrate that *Jack‐of‐all‐trades* species (such as *Typha latifolia* and *Lythrum salicaria*) had little modification of their trait distribution along the diversity gradient. The stability of both density and trait distribution suggests that the species exploit the same resources without losing their demographic advantages. Therefore, species diversity may increase because of the addition of species with niche distant from the *Jack‐of‐all‐trades* species (as suggested by the increased dispersion with species richness in most traits). Conversely, *Master‐of‐some* strategy is characterized by species where intraspecific variation is related to demography. In response to species richness, we show that the decrease in population density was associated with an increased dispersion of leaf angle and LDMC values. This pattern suggest that *Master‐of‐some* individuals present in a more diverse community multiplied the ways to exploit light and nutrient resources. Therefore, demographic advantage of *Master‐of‐some* species may decrease along the diversity gradient, freeing niche space for new species.

By showing the interactions of intraspecific variation, biotic selection, and demographic patterns, we highlighted the role that selection for individuals plays in community assembly. In doing so, the concepts underpinning community and population ecology are brought closer together. Hennion et al. (2016) showed that species richness persistently altered the amine metabolic profile of a grassland species. Meilhac et al. (2020) showed that coexistence of clonal species over a short period of time was dependent upon both genotype selection and individual‐level intraspecific trait variation. Waterway et al. (2016) demonstrated that competitive interactions have driven the historical diversification of coexisting sedge species in fens. While it is regularly argued that the selection of individuals within a community is of evolutionary importance (Post & Palkovacs, 2009), the joint study of population and community levels is rarely combined in community ecology (Maire et al., 2013; Salguero‐Gómez et al., 2018). Here, we show that using a hierarchical approach along an abiotic and an independent biotic gradient, we are able to better understand how traits vary across biological levels.

## 5 CONCLUSION

Disentangling selection processes across levels of biological organization and considering a rich set of traits representing different trait space dimensions revealed the simultaneous pressures acting on individuals. By focusing on individuals, we were able to show that (1) plant species richness was a major driver of trait selection within community, (2) species adapted to the above gradient by mainly modifying their space and light acquisition traits, and (3) trait intraspecific variation was related to demographic strategies. Finally, our study highlights the importance of intraspecific trait variation and individual selection for community assembly, revealing the potential evolutionary consequences of local biotic gradients.

## AUTHOR CONTRIBUTIONS

**Lucas Deschamps:** Conceptualization (equal); data curation (lead); formal analysis (lead); methodology (lead); resources (equal); software (lead); validation (lead); visualization (lead); writing – original draft (lead). **Raphaël Proulx:** Conceptualization (equal); funding acquisition (equal); methodology (supporting); supervision (equal); visualization (supporting); writing – review and editing (equal). **Nicolas Gross:** Conceptualization (supporting); supervision (supporting); writing – review and editing (equal). **Christopher J. Watson:** Conceptualization (supporting); writing – review and editing (supporting). **Guillaume Rheault:** Formal analysis (supporting); investigation (supporting); methodology (supporting); resources (supporting); writing – review and editing (supporting). **Vincent Maire:** Conceptualization (equal); funding acquisition (equal); investigation (lead); methodology (equal); project administration (lead); resources (equal); supervision (equal); validation (equal); visualization (supporting); writing – review and editing (lead).

## CONFLICT OF INTEREST STATEMENT

There is no conflict of interest to declare.

### OPEN RESEARCH BADGES

This article has earned Open Data and Open Materials badges. Data and materials are available at https://doi.org/10.5683/SP3/JFFQ0W.

## Supporting information


Appendix S1

## Data Availability

Data will be accessible on the Borealis data repository and can be quoted as: Maire et al. (2023, https://doi.org/10.5683/SP3/JFFQ0W).

## References

[ece39959-bib-0001] Agati, G. , Cerovic, Z. G. , Pinelli, P. , & Tattini, M. (2011). Light‐induced accumulation of ortho‐dihydroxylated flavonoids as non‐destructively monitored by chlorophyll fluorescence excitation techniques. Environmental and Experimental Botany, 73, 3–9.

[ece39959-bib-0002] Anderegg, L. D. L. (2023). Why can't we predict traits from the environment? New Phytologist, 237, 1998–2004. 10.1111/nph.18586 36308517

[ece39959-bib-0003] Anderegg, L. D. L. , Berner, L. T. , Badgley, G. , Sethi, M. L. , Law, B. E. , & HilleRisLambers, J. (2018). Within‐species patterns challenge our understanding of the leaf economics spectrum. Ecology Letters, 21, 734–744. 29569818 10.1111/ele.12945

[ece39959-bib-0004] Austin, M. P. , & Smith, T. M. (1989). A new model for the continuum concept. Vegetatio, 83, 35–47.

[ece39959-bib-0005] Berdugo, M. , Maestre, F. T. , Kéfi, S. , Gross, N. , Le Bagousse‐Pinguet, Y. , & Soliveres, S. (2019). Aridity preferences alter the relative importance of abiotic and biotic drivers on plant species abundance in global drylands. Journal of Ecology, 107(1), 190–202.

[ece39959-bib-0006] Bittebiere, A. K. , Saiz, H. , & Mony, C. (2018). New insights from multidimensional trait space responses to competition in two clonal plant species. Functional Ecology, 33(2), 297–307.

[ece39959-bib-0007] Bjorkman, A. , Myers‐Smith, I. H. , Elmendorf, S. C. , Normand, S. , Rüger, N. , Beck, P. , Blach‐Overgaard, A. , Blok, D. , Cornelissen, J. H. C. , Forbes, B. C. , Georges, D. , Goetz, S. J. , Guay, K. C. , Henry, G. H. R. , HilleRisLambers, J. , Hollister, R. D. , Karger, D. N. , Kattge, J. , Manning, P. , … Weiher, E. (2018). Plant functional trait change across a warming tundra biome. Nature, 562(7725), 57–62. 30258229 10.1038/s41586-018-0563-7

[ece39959-bib-0008] Brouillet, L. , Hay, S. G. , Goulet, I. , & Marie‐Victorin, S. (2002). Flore laurentienne (3e édition ed., p. 1112). Éditions Gaëtan Morin.

[ece39959-bib-0009] Bürkner, P. C. (2017). Brms: An R package for Bayesian multilevel models using Stan. Journal of Statistical Software, 80, 1–28.

[ece39959-bib-0010] Cavender‐Bares, J. , Kitajima, K. , & Bazzaz, F. A. (2004). Multiple trait associations in relation to habitat differentiation among 17 Floridian oak species. Ecological Monographs, 74, 635–662.

[ece39959-bib-0011] Cepeda‐Cuervo, E. (2015). Beta regression models: Joint mean and variance modeling. Journal of Statistical Theory and Practice, 9, 134–145.

[ece39959-bib-0012] Chalmandrier, L. , Münkemüller, T. , Colace, M. P. , Renaud, J. , Aubert, S. , Carlson, B. Z. , Clément, J. C. , Legay, N. , Pellet, G. , Saillard, A. , Lavergne, S. , & Thuiller, W. (2017). Spatial scale and intraspecific trait variability mediate assembly rules in alpine grasslands. Journal of Ecology, 105, 277–287.

[ece39959-bib-0013] Chesson, P. (2000). Mechanisms of maintenance of species diversity. Annual Review of Ecology and Systematics, 31, 343–366.

[ece39959-bib-0014] Cornwell, W. K. , & Ackerly, D. D. (2009). Community assembly and shifts in plant trait distributions across an environmental gradient in coastal California. Ecological Monographs, 79(1), 109–126.

[ece39959-bib-0015] Cornwell, W. K. , Schwilk, D. W. , & Ackerly, D. D. (2006). A trait‐based test for habitat filtering: Convex hull volume. Ecology, 87, 1465–1471. 16869422 10.1890/0012-9658(2006)87[1465:attfhf]2.0.co;2

[ece39959-bib-0016] Da Silveira Pontes, L. , Maire, V. , Schellberg, J. , & Louault, F. (2015). Grass strategies and grassland community responses to environmental drivers: A review. Agronomy for Sustainable Development, 35, 1297–1318.

[ece39959-bib-0017] Davrinche, A. , & Haider, S. (2021). Intra‐specific leaf trait responses to species richness at two different local scales. Basic and Applied Ecology, 55, 20–32.

[ece39959-bib-0018] De Bello, F. , Lavorel, S. , Lavergne, S. , Albert, C. H. , Boulangeat, I. , Mazel, F. , & Thuiller, W. (2013). Hierarchical effects of environmental filters on the functional structure of plant communities: A case study in the French Alps. Ecography, 36, 393–402.

[ece39959-bib-0019] De Bello, F. , Price, J. N. , Münkemuüller, J. , Liira, J. , Zobel, M. , Thuiller, W. , Gerhold, P. , Götzenberger, L. , Lavergne, S. , Leps, J. , Zobel, K. , & Pärtel, M. (2012). Functional species pool framework to test for biotic effects on community assembly functional species pool framework to test for biotic effects on community assembly. Ecology, 93, 2263–2273. 23185887 10.1890/11-1394.1

[ece39959-bib-0020] Dong, N. , Prentice, I. C. , Wright, I. J. , Evans, B. J. , Togashi, H. F. , Caddy‐Retalic, S. , McInerney, F. A. , Sparrow, B. , Leitch, E. , & Lowe, A. J. (2020). Components of leaf‐trait variation along environmental gradients. New Phytologist, 228, 82–94. 32198931 10.1111/nph.16558

[ece39959-bib-0021] Elzinga, C. L. , Salzer, D. W. , & Willoughby, J. W. (1998). Measuring and monitoring plant populations. Technical Reference 1730‐1. USDI, BLM.

[ece39959-bib-0022] Gommers, C. M. M. , Visser, E. J. W. , St Onge, K. R. , Voesenek, L. A. C. J. , & Pierik, R. (2013). Shade tolerance: When growing tall is not an option. Trends in Plant Science, 18(2), 65–71. 23084466 10.1016/j.tplants.2012.09.008

[ece39959-bib-0023] Grime, J. P. (1977). Evidence for the existence of three primary strategies in plants and its relevance to ecological and evolutionary theory. The American Naturalist, 111, 1169–1194.

[ece39959-bib-0024] Grime, J. P. (2006). Trait convergence and trait divergence in herbaceous plant communities: Mechanisms and consequences. Journal of Vegetation Science, 17, 255–260.

[ece39959-bib-0025] Gross, N. , Börger, L. , Duncan, R. P. , & Hulme, P. E. (2013). Functional differences between alien and native species: Do biotic interactions determine the functional structure of highly invaded grasslands? Functional Ecology, 27, 1262–1272.

[ece39959-bib-0026] Gross, N. , Börger, L. , Soriano‐Morales, S. I. , Le Bagousse‐Pinguet, Y. , Quero, J. L. , García‐Gómez, M. , Valencia‐Gómez, E. , & Maestre, F. T. (2013). Uncovering multiscale effects of aridity and biotic interactions on the functional structure of Mediterranean shrublands. Journal of Ecology, 101(3), 637–649.

[ece39959-bib-0027] Gubsch, M. , Roscher, C. , Gleixner, G. , Habekost, M. , Lipowsky, A. , Schmid, B. , Schulze, E. D. , Steinbeiss, S. , & Buchmann, N. (2011). Foliar and soil δ15N values reveal increased nitrogen partitioning among species in diverse grassland communities. Plant, Cell and Environment, 34, 895–908. 10.1111/j.1365-3040.2011.02287.x 21332507

[ece39959-bib-0028] Hart, S. P. , Schreiber, S. J. , Levine, J. M. , & Coulson, T. (2016). How variation between individuals affects species coexistence. Ecology Letters, 19, 825–838. 27250037 10.1111/ele.12618

[ece39959-bib-0029] Hennion, F. , Litrico, I. , Bartish, I. V. , Weigelt, A. , Bouchereau, A. , & Prinzing, A. (2016). Ecologically diverse and distinct neighbourhoods trigger persistent phenotypic consequences, and amine metabolic profiling detects them. Journal of Ecology, 104, 125–137.

[ece39959-bib-0030] Hikosaka, K. , & Hirose, T. (1997). Leaf angle as a strategy for light competition: Optimal and evolutionarily stable light‐extinction coefficient within a leaf canopy. Ecoscience, 4, 501–507.

[ece39959-bib-0031] HilleRisLambers, J. , Adler, P. B. , Harpole, W. S. , Levine, J. M. , & Mayfield, M. M. (2012). Rethinking community assembly through the lens of coexistence theory. Annual Review of Ecology, Evolution and Systematics, 43, 227–248.

[ece39959-bib-0032] Hodgson, J. G. , Montserrat‐Martí, G. , Charles, M. , Jones, G. , Wilson, P. , Shipley, B. , Sharafi, M. , Cerabolini, B. E. L. , Cornelissen, J. H. C. , Band, S. R. , Bogard, A. , Castro‐Díez, P. , Guerrero‐Campo, J. , Palmer, C. , Pérez‐Rontomé, M. C. , Carter, G. , Hynd, A. , Romo‐Díez, A. , de Torres Espuny, L. , & Royo Pla, F. (2011). Is leaf dry matter content a better predictor of soil fertility than specific leaf area? Annals of Botany, 108, 1337–1345. 21948627 10.1093/aob/mcr225 PMC3197453

[ece39959-bib-0033] Hulshof, C. M. , Violle, C. , Spasojevic, M. J. , Mcgill, B. , Damschen, E. , Harrison, S. , & Enquist, B. J. (2013). Intra‐specific and inter‐specific variation in specific leaf area reveal the importance of abiotic and biotic drivers of species diversity across elevation and latitude. Journal of Vegetation Science, 24, 921–931.

[ece39959-bib-0034] Huston, M. A. (2014). Disturbance, productivity, and species diversity: Empiricism vs. logic in ecological theory. Ecology, 95(9), 2382–2396.

[ece39959-bib-0035] Izaguirre, M. M. , Mazza, C. A. , Svatos, A. , Baldwin, I. T. , & Ballare, C. L. (2007). Solar ultraviolet‐B radiation and insect herbivory trigger partially overlapping phenolic responses in *Nicotiana attenuata* and *Nicotiana longiflora* . Annals of Botany, 99, 103–109. 17210605 10.1093/aob/mcl226 PMC2802969

[ece39959-bib-0036] Jager, M. M. , Richardson, S. J. , Bellingham, P. J. , Clearwater, M. J. , & Laughlin, D. C. (2015). Soil fertility induces coordinated responses of multiple independent functional traits. Journal of Ecology, 103, 374–385.

[ece39959-bib-0037] Kull, O. , & Niinemets, Ü. (1998). Distribution of leaf photosynthetic properties in tree canopies: Comparison of species with different shade tolerance. Functional Ecology, 12, 472–479.

[ece39959-bib-0038] Kunstler, G. , Lavergne, S. , Courbaud, B. , Thuiller, W. , Vieilledent, G. , Zimmermann, N. E. , Kattge, J. , & Coomes, D. A. (2012). Competitive interactions between forest trees are driven by species' trait hierarchy, not phylogenetic or functional similarity: Implications for forest community assembly. Ecology Letters, 15, 831–840. 22625657 10.1111/j.1461-0248.2012.01803.x PMC4003531

[ece39959-bib-0039] Laughlin, D. C. , & Joshi, C. (2015). Theoretical consequences of trait‐based environmental filtering for the breadth and shape of the niche: New testable hypotheses generated by the Traitspace model. Ecological Modelling, 307, 10–21.

[ece39959-bib-0040] Le Bagousse‐Pinguet, Y. , Börger, L. , Quero, J. L. , Garcia‐Gomez, M. , Soriano, S. , Maestre, F. T. , & Gross, N. (2015). Traits of neighbouring plants and space limitation determine intraspecific trait variability in semi‐arid shrublands. Journal of Ecology, 103, 1647–1657.

[ece39959-bib-0041] Le Bagousse‐Pinguet, Y. , de Bello, F. , Vandewalle, M. , Leps, J. , & Sykes, M. T. (2014). Species richness of limestone grasslands increases with trait overlap: Evidence from within‐ and between‐species functional diversity partitioning. Journal of Ecology, 102, 466–474.

[ece39959-bib-0042] Le Bagousse‐Pinguet, Y. , Gross, N. , Maestre, F. T. , Maire, V. , de Bello, F. , Fonseca, C. R. , Kattge, J. , Valencia, E. , Leps, J. , & Liancourt, P. (2017). Testing the environmental filtering concept in global drylands. Journal of Ecology, 105(4), 1058–1069. 28642625 10.1111/1365-2745.12735 PMC5476209

[ece39959-bib-0043] Levine, J. M. , & HilleRisLambers, J. (2009). The importance of niches for the maintenance of species diversity. Nature, 461, 254–257. 19675568 10.1038/nature08251

[ece39959-bib-0044] Lortie, C. J. , Brooker, R. W. , Choler, P. , Kikvidze, Z. , Michalet, R. , & Pugnaire, F. I. (2004). Rethinking plant community theory edited by Foxit reader. Oikos, 107, 433–438.

[ece39959-bib-0045] Maire, V. , Deschamps, L. , & Proulx, R. (2023). Plant functional traits measured at the individual level within four temperate wetland types (bog, fen, meadow, and marsh) in North‐East America. *Borealis*, DRAFT VERSION 10.5683/SP3/JFFQ0W

[ece39959-bib-0046] Maire, V. , Gross, N. , Börger, L. , Proulx, R. , Wirth, C. , Da Silveira, P. L. , Soussana, J. F. , & Louault, F. (2012). Habitat filtering and niche differentiation jointly explain species relative abundance within grassland communities along fertility and disturbance gradients. New Phytologist, 196, 497–509. 22931515 10.1111/j.1469-8137.2012.04287.x

[ece39959-bib-0047] Maire, V. , Soussana, J. F. , Gross, N. , Bachelet, B. , Pagès, L. , Martin, R. , Reinhold, T. , Wirth, C. , & Hill, D. (2013). Plasticity of plant form and function sustains productivity and dominance along environment and competition gradients. A modeling experiment with GEMINI. Ecological Modelling, 254(10), 80–91.

[ece39959-bib-0048] Mattila, H. , Valev, D. , Havurinne, V. , Khorobrykh, S. , Virtanen, O. , Antinluoma, M. , Mishra, K. B. , & Tyystjärvi, E. (2018). Degradation of chlorophyll and synthesis of flavonols during autumn senescence‐the story told by individual leaves. AoB Plants, 10, 1–13. 10.1093/aobpla/ply028 PMC6007487 29977486

[ece39959-bib-0049] Mayfield, M. M. , & Levine, J. M. (2010). Opposing effects of competitive exclusion on the phylogenetic structure of communities. Ecology Letters, 13, 1085–1093. 20576030 10.1111/j.1461-0248.2010.01509.x

[ece39959-bib-0050] McKane, R. B. , Johnson, L. C. , Shaver, G. R. , Nadelhoffer, K. J. , Rastetter, E. B. , Fry, B. , Giblin, A. E. , Kielland, K. , Kwlatkowski, B. L. , Laundre, J. A. , & Murray, G. (2002). Resource‐based niches provide a basis for plant species diversity and dominance in arctic tundra. Nature, 415, 68–71. 11780117 10.1038/415068a

[ece39959-bib-0051] Meilhac, M. , Deschamps, L. , Maire, V. , Flajoulot, S. , & Litrico, I. (2020). Both selection and plasticity drive niche differentiation in experimental grasslands. Nature Plants, 6, 28–33. 31873193 10.1038/s41477-019-0569-7

[ece39959-bib-0052] Messier, J. , McGill, B. J. , Enquist, B. J. , & Lechowicz, M. J. (2017). Trait variation and integration across scales: Is the leaf economic spectrum present at local scales? Ecography, 40, 685–697.

[ece39959-bib-0053] Millenaar, F. F. , Van Zanten, M. , Cox, M. C. H. , Pierik, R. , Voesenek, L. A. C. J. , & Peeters, A. M. (2009). Differential petiole growth in Arabidopsis thaliana: Photocontrol and hormonal regulation. New Phytologist, 184(1), 141–152. 19558423 10.1111/j.1469-8137.2009.02921.x

[ece39959-bib-0054] Pérez‐Harguindeguy, N. , Diaz, S. , Garnier, E. , Lavorel, S. , Poorter, H. , Jaureguiberry, P. , Bret‐Harte, M. S. S. , Cornwell, W. K. K. , Craine, J. M. M. , Gurvich, D. E. E. , Urcelay, C. , Veneklaas, E. J. , Reich, P. B. , Poorter, L. , Wright, I. J. , Ray, P. , Enrico, L. , Pausas, J. G. , De Vos, A. C. , & Buchmann, N. (2013). New handbook for standardized measurment of plant functional traits worldwide. Australian Journal of Botany, 61, 167–234.

[ece39959-bib-0055] Poorter, H. , Niklas, K. J. , Reich, P. B. , Oleksyn, J. , Poot, P. , & Mommer, L. (2012). Biomass allocation to leaves, stems and roots: Meta‐analyses of interspecific variation and environmental control. New Phytologist, 193, 30–50. 22085245 10.1111/j.1469-8137.2011.03952.x

[ece39959-bib-0056] Post, D. M. , & Palkovacs, E. P. (2009). Eco‐evolutionary feedbacks in community and ecosystem ecology: Interactions between the ecological theatre and the evolutionary play. Philosophical Transactions of the Royal Society B: Biological Sciences, 364, 1629–1640. 10.1098/rstb.2009.0012 PMC2690506 19414476

[ece39959-bib-0057] Rheault, G. , Proulx, R. , & Bonin, L. (2015). Plant species richness prolongs the growing season of freely assembled riparian herbaceous communities under dry climatic conditions. Agriculture, Ecosystems & Environment, 200, 71–78.

[ece39959-bib-0058] Richards, C. L. , Bossdorf, O. , Muth, N. Z. , Gurevitch, J. , & Pigliucci, M. (2006). Jack of all trades, master of some? On the role of phenotypic plasticity in plant invasions. Ecology Letters, 9, 981–993. 16913942 10.1111/j.1461-0248.2006.00950.x

[ece39959-bib-0059] Rigby, R. A. , & Stasinopoulos, D. M. (2005). Generalized additive models for location, scale and shape. Applied Statistics, 54, 507–554.

[ece39959-bib-0060] Salguero‐Gómez, R. , Violle, C. , Gimenez, O. , & Childs, D. (2018). Delivering the promises of trait‐based approaches to the needs of demographic approaches, and vice versa. Functional Ecology, 32, 1424–1435. 30034074 10.1111/1365-2435.13148 PMC6049886

[ece39959-bib-0061] Santamaria, L. , Figuerola, J. , Pilon, J. J. , Mjelde, M. , Green, A. J. , de Boer, T. , King, R. A. , & Gornall, R. J. (2003). Plant performance across latitude: The role of plasticity and local adaptation in an aquatic plant. Ecology, 84(9), 2454–2461.

[ece39959-bib-0062] Scogings, P. F. (2018). Foliar flavonol concentration in Sclerocarya birrea saplings responds to nutrient fertilisation according to growth‐differentiation balance hypothesis. Phytochemistry Letters, 23, 180–184.

[ece39959-bib-0063] Shipley, B. (2010). From plant traits to vegetation structure. Cambridge Universty Press.

[ece39959-bib-0064] Shipley, B. , Vile, D. , & Garnier, E. (2006). From plant traits to plant communities: A statistical mechanistic approach to biodiversity. Science, 314, 812–814. 17023613 10.1126/science.1131344

[ece39959-bib-0065] Siebenkäs, A. , Schumacher, J. , & Roscher, C. (2017). Trait variation in response to resource availability and plant diversity modulates functional dissimilarity among species in experimental grasslands. Journal of Plant Ecology, 10(6), 981–993.

[ece39959-bib-0066] Siefert, A. , Fridley, J. D. , & Ritchie, M. E. (2014). Community functional responses to soil and climate at multiple spatial scales: When does intraspecific variation matter? PLoS One, 9(10), e111189. 25329794 10.1371/journal.pone.0111189 PMC4203824

[ece39959-bib-0067] Smyth, G. K. (1989). Generalized linear models with varying dispersion. Journal of the Royal Statistical Society. Series B (Methodological), 51, 47–60.

[ece39959-bib-0068] Spasojevic, M. J. , & Suding, K. N. (2012). Inferring community assembly mechanisms from functional diversity patterns: The importance of multiple assembly processes. Journal of Ecology, 100(3), 652–661.

[ece39959-bib-0069] Umaña, M. N. , & Swenson, N. G. (2019). Does trait variation within broadly distributed species mirror patterns across species? A case study in Puerto Rico. Ecology, 100(8), e02745. 31032887 10.1002/ecy.2745

[ece39959-bib-0070] Violle, C. , Enquist, B. J. , McGill, B. J. , Jiang, L. , Albert, C. H. , Hulshof, C. , Jung, V. , & Messier, J. (2012). The return of the variance: Intraspecific variability in community ecology. Trends in Ecology and Evolution, 27, 244–252. 22244797 10.1016/j.tree.2011.11.014

[ece39959-bib-0071] Violle, C. , Navas, M. L. , Vile, D. , Kazakou, E. , Fortunel, C. , Hummel, I. , & Garnier, E. (2007). Let the concept of trait be functional! Oikos, 116, 882–892.

[ece39959-bib-0072] Waterway, M. J. , Martins, K. T. , Dabros, A. , Prado, A. , & Lechowicz, M. J. (2016). Ecological and evolutionary diversification within the genus Carex (Cyperaceae): Consequences for community assembly in subarctic fens. Systematic Botany, 41, 558–579.

[ece39959-bib-0073] Weiher, E. , & Keddy, P. A. (1995). The assembly of experimental wetland communities. Oikos, 73, 323–335.

[ece39959-bib-0074] Weiner, J. , & Thomas, S. C. (1986). Size variability and competition in plant monocultures. Oikos, 47, 211–222.

[ece39959-bib-0075] Wiktor, K. , & van Diggelen, R. (2004). Light as an environmental filter in fen vegetation. Journal of Vegetation Science, 15, 583–594.

[ece39959-bib-0076] Willis, K. J. , & Whittaker, R. J. (2002). Species diversity – Scale matters. Science, 295, 1245–1248. 11847328 10.1126/science.1067335

[ece39959-bib-0077] Wolf, A. A. , Funk, J. L. , Selmants, P. C. , Morozumi, C. N. , Hernándeze, D. L. , Pasari, J. R. , & Zavaleta, E. S. (2021). Trait‐based filtering mediates the effects of realistic biodiversity losses on ecosystem functioning. Proceedings of the National Academy of Sciences of the United States of America, 118(26), e2022757118. 34162704 10.1073/pnas.2022757118 PMC8256034

[ece39959-bib-0078] Wright, I. J. , Reich, P. B. , Westoby, M. , Ackerly, D. D. , Baruch, Z. , Bongers, F. , Cavender‐bares, J. , Chapin, T. , Cornelissen, J. H. C. , Diemer, M. , Flexas, J. , Garnier, E. , Groom, P. K. , & Gulias, J. (2004). The worldwide leaf economics spectrum. Nature, 428, 821–827. 15103368 10.1038/nature02403

[ece39959-bib-0079] Yao, Y. , Vehtari, A. , Simpson, D. , & Gelman, A. (2017). Using stacking to average Bayesian predictive distributions. Bayesian Analysis, 13(3), 917–1003.

[ece39959-bib-0080] Zuppinger‐Dingley, D. , Schmid, B. , Petermann, J. S. , Yadav, V. , De Deyn, G. B. , & Flynn, D. F. B. (2014). Selection for niche differentiation in plant communities increases biodiversity effects. Nature, 515, 108–111. 25317555 10.1038/nature13869

