# Supplementary material for: Species richness drives selection of individuals within wetlands based on traits related to acquisition and utilization of light
Source: Ecol Evol. 2023 Apr 7;13(4):e9959. doi: 10.1002/ece3.9959 (PMC10082176; doi:10.1002/ece3.9959)
Supplement: Supplementary file 1 — Appendix S1 [file ECE3-13-e9959-s001.docx]

**Supplementary materials for**

**Species richness drove selection of individuals within wetlands based on traits related to acquisition and utilization of light**

Lucas Deschamps, Raphaël Proulx, Guillaume Rheault, Nicolas Gross, Christopher Watson, Vincent Maire

**Summary**

[A. Site and species observations 2](#_Toc125994745)

[B. Bayesian hierarchical distributional modelling 5](#_Toc125994746)

[a. Framework 5](#_Toc125994747)

[b. Distributional modelling 5](#_Toc125994748)

[i. Gamma distribution 6](#_Toc125994749)

[ii. Beta distribution 6](#_Toc125994750)

[c. Hierarchical model formulation 7](#_Toc125994751)

[d. Model diagnosis 8](#_Toc125994752)

[e. Model predictions 9](#_Toc125994753)

[i. Ecosystem level predictions 9](#_Toc125994754)

[ii. Community level predictions 12](#_Toc125994755)

[iii. Species level predictions 20](#_Toc125994756)

[f. Model diagnosis 28](#_Toc125994757)

[i. Community level 28](#_Toc125994758)

[ii. Species level 34](#_Toc125994759)

[C. Literature cited 42](#_Toc125994760)

1. Site and species observations


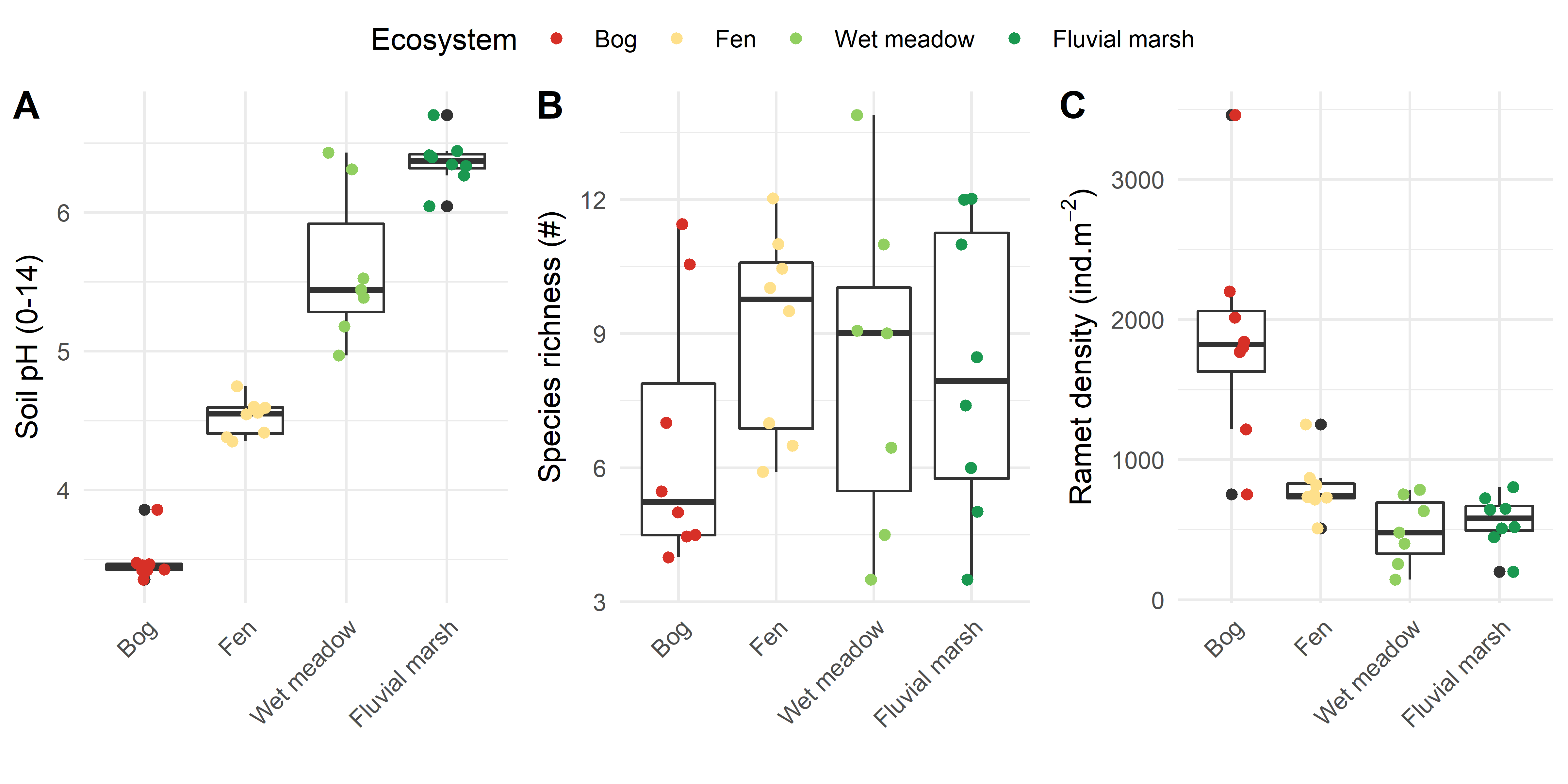


**Figure S1**: Abiotic and biotic characteristics of plots in each ecosystem. Panels a, b c present the between ecosystems differences in soil pH, species richness and ramet density, respectively.


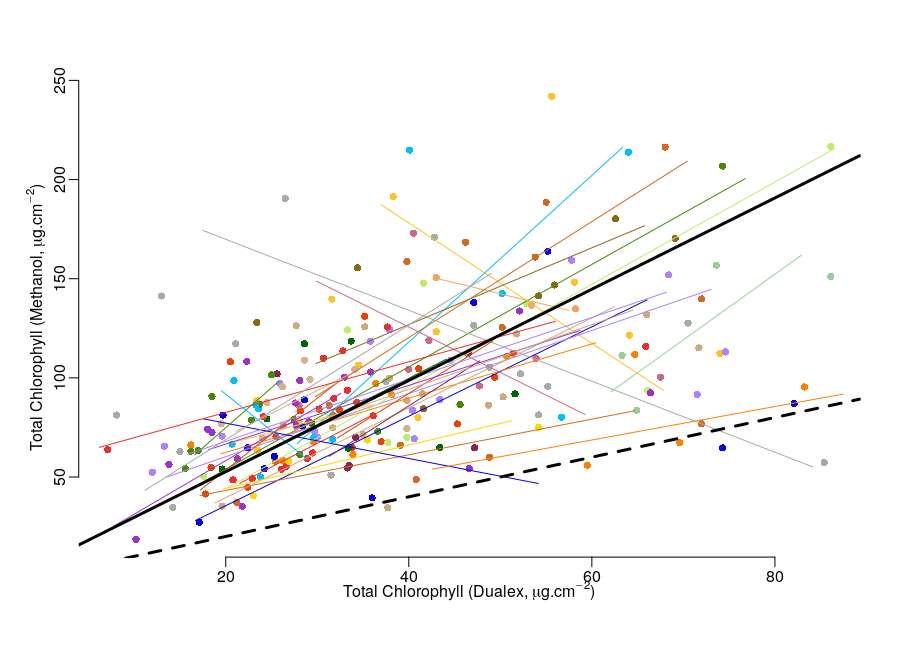


**Figure S2**: Relationship between total chlorophyll concentration extracted with methanol and measured spectrophotometrically and superficial chlorophyll estimated using Dualex device. Plain line represents the global relationship, each colored line represent relationship for each tested species, and dashed line represents a 1:1 relationship.


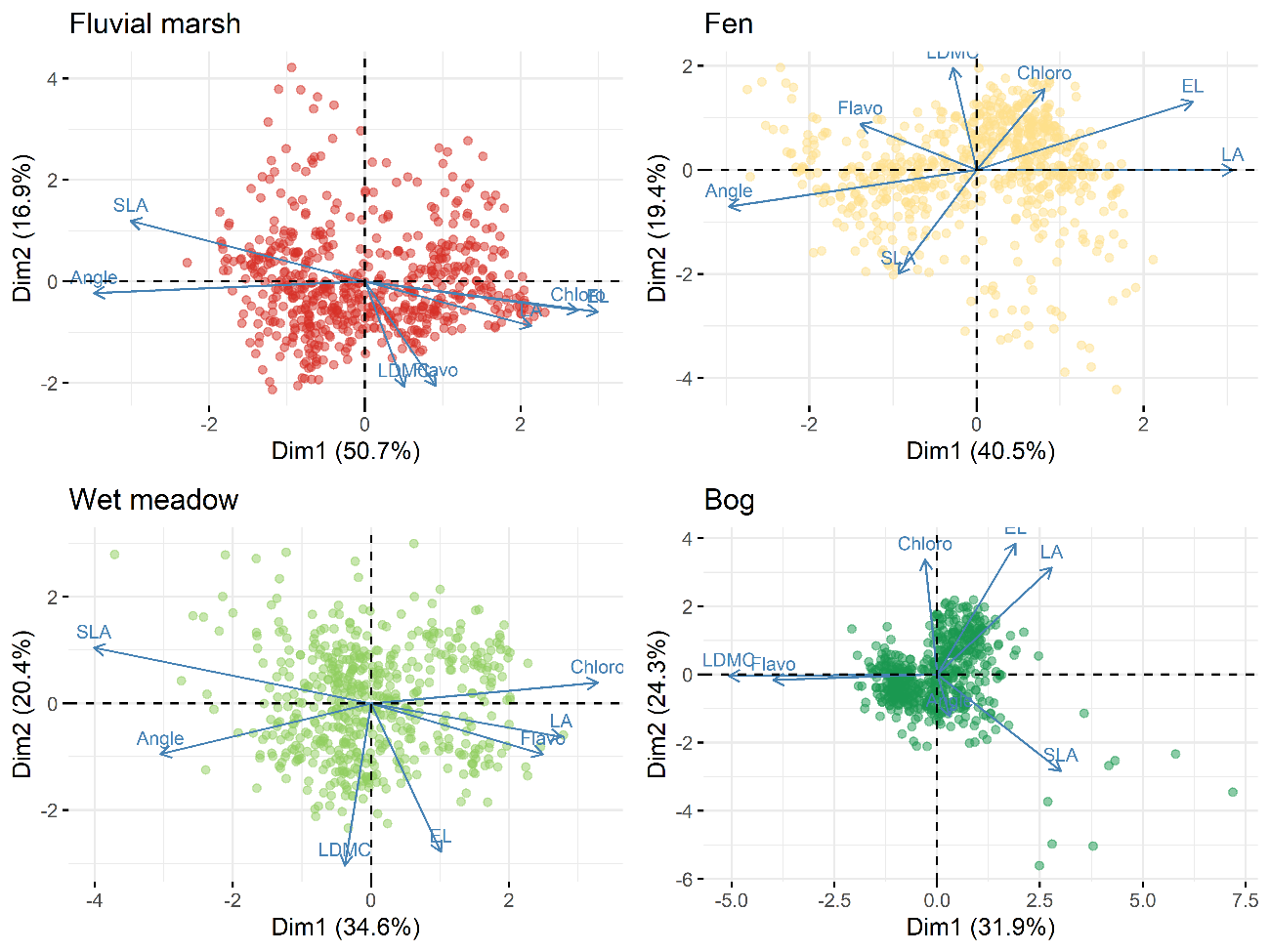


**Figure S3**: Principal component analysis of trait values within each ecosystems. PCA axes were rotated using varimax operation.

**Table S1:** Plant traits of wetland species. Angle: leaf angle; Chlo.: leaf chlorophyll content; EL: extended length; Flav.: leaf flavonoid content; LA: leaf area; LDMC: leaf dry matter content; SLA: specific leaf area

| **Species** | **EL**  (cm) | **Angle**  (°) | **LA**  (cm^2^) | **SLA**  (cm^2^ g^-1^) | **LDMC**  (g g^-1^) | **Flav.**  (μg cm^-2^) | **Chlo.**  (μg cm^-2^) |
| --- | --- | --- | --- | --- | --- | --- | --- |
| *Acorus calamus* | 85.9 | 17.3 | 49.40 | 124.1 | 0.240 | 1.72 | 54.5 |
| *Alnus rugosa* | 52.7 | 53.0 | 9.57 | 153.0 | 0.404 | 1.92 | 28.1 |
| *Andromeda polifolia var. latifolia* | 18.3 | 22.2 | 0.87 | 58.8 | 0.483 | 1.73 | 49.3 |
| *Betula pumila* | 53.6 | 25.7 | 4.12 | 126.0 | 0.405 | 1.79 | 27.5 |
| *Bolboschoenus fluviatilis* | 82.3 | 19.8 | 37.80 | 180.4 | 0.259 | 1.20 | 34.1 |
| *Butomus umbellatus* | 68.9 | 9.6 | 29.20 | 165.3 | 0.129 | 1.05 | 55.1 |
| *Calamagrostis canadensis* | 68.8 | 31.4 | 10.50 | 269.2 | 0.364 | 1.12 | 34.7 |
| *Carex cf. echinata* | 41.5 | 15.0 | 3.89 | 162.1 | 0.400 | 0.50 | 24.4 |
| *Carex crinita* | 85.8 | 13.0 | 24.40 | 336.1 | 0.320 | 0.92 | 21.3 |
| *Carex lasiocarpa* | 68.2 | 6.5 | 6.72 | 89.4 | 0.401 | 1.61 | 50.6 |
| *Carex oligosperma* | 63.8 | 6.6 | 6.47 | 90.0 | 0.407 | 1.56 | 42.1 |
| *Carex sect. Phacocystis* | 73.2 | 6.0 | 18.00 | 170.4 | 0.412 | 1.55 | 36.5 |
| *Carex subgen. vignea* | 45.2 | 10.0 | 4.14 | 201.9 | 0.335 | 0.84 | 41.5 |
| *Carex vesicaria* | 86.9 | 6.7 | 29.90 | 213.8 | 0.303 | 1.01 | 39.8 |
| *Chamaedaphne calyculata* | 17.4 | 17.3 | 1.17 | 106.3 | 0.507 | 1.91 | 38.9 |
| *Comarum palustre* | 43.2 | 39.1 | 60.90 | 198.7 | 0.274 | 1.40 | 33.2 |
| *Drosera rotundifolia* | 3.72 | 49.5 | 0.33 | 371.3 | 0.165 | 1.28 | 14.9 |
| *Dulichium arundinaceum* | 51.8 | 49.1 | 2.56 | 277.1 | 0.365 | 1.48 | 39.0 |
| *Eleocharis sp.* | 49.6 | 5.6 | 5.90 | 135.0 | 0.309 | 1.34 | 49.6 |
| *Equisetum fluviatile* | 81.9 | 28.0 | 6.96 | 132.8 | 0.207 | 0.81 | 44.8 |
| *Eriophorum gracile* | 59.3 | 10.3 | 13.80 | 104.3 | 0.368 | 1.66 | 48.4 |
| *Eriophorum tenellum* | 41.8 | 13.5 | 3.23 | 123.3 | 0.313 | 1.50 | 55.6 |
| *Eriophorum vaginatum ssp. spissum* | 43.1 | 7.3 | 3.52 | 106.7 | 0.396 | 1.35 | 63.6 |
| *Eriophorum virginicum* | 29.4 | 18.3 | 2.86 | 95.5 | 0.362 | 1.41 | 57.5 |
| *Eurybia maculata* | 110 | 68.0 | 11.70 | 199.6 | 0.249 | 2.04 | 27.5 |
| *Gallium palustre* | 45 | 69.1 | 0.86 | 851.6 | 0.103 | 0.40 | 25.8 |
| *Gaylussacia baccata* | 23.4 | 17.2 | 2.52 | 166.3 | 0.408 | 1.93 | 24.5 |
| *Glyceria canadensis* | 71.5 | 20.7 | 6.56 | 170.1 | 0.381 | 1.58 | 21.3 |
| *Glyceria grandis* | 83.3 | 18.5 | 45.50 | 235.4 | 0.249 | 1.10 | 28.4 |
| *Hypericum virginicum* | 37.4 | 38.8 | 5.95 | 522.7 | 0.326 | 1.31 | 20.1 |
| *Impatiens capensis* | 39.9 | 68.8 | 5.44 | 822.1 | 0.159 | 0.61 | 17.5 |
| *Iris versicolor* | 49.6 | 10.4 | 35.30 | 131.1 | 0.208 | 1.54 | 43.0 |
| *Kalmia angustifolia* | 21.8 | 35.4 | 1.53 | 101.1 | 0.490 | 2.12 | 33.6 |
| *Kalmia polifolia* | 17.9 | 20.1 | 0.57 | 93.1 | 0.412 | 1.80 | 33.0 |
| *Lathyrus palustris* | 74.3 | 46.7 | 9.41 | 352.6 | 0.255 | 1.26 | 31.8 |
| *Leersia oryzoides* | 106 | 64.0 | 7.65 | 196.4 | 0.433 | 1.27 | 20.2 |
| *Lycopus europaeus* | 37.6 | 52.0 | 4.68 | 851.3 | 0.157 | 0.46 | 15.7 |
| *Lysimachia terrestris* | 40.1 | 37.3 | 2.60 | 196.2 | 0.284 | 1.87 | 30.6 |
| *Lysimachia thyrsiflora* | 53.9 | 45.0 | 13.30 | 401.0 | 0.180 | 1.28 | 28.7 |
| *Lythrum salicaria* | 60 | 48.7 | 5.99 | 368.3 | 0.222 | 1.02 | 26.0 |
| *Oclemena nemoralis* | 27.8 | 39.5 | 0.94 | 236.6 | 0.328 | 1.57 | 26.8 |
| *Onoclea sensibilis* | 47.5 | 27.6 | 100.00 | 237.8 | 0.293 | 1.14 | 21.3 |
| *Phalaris arundinacea* | 87.9 | 43.8 | 17.80 | 363.2 | 0.266 | 0.75 | 32.6 |
| *Platanthera blephariglottis* | 11.4 | 32.0 | 5.23 | 297.8 | 0.113 | 0.77 | 15.7 |
| *Pogonia ophioglossoides* | 22.2 | 16.0 | 15.10 | 267.0 | 0.128 | 1.48 | 21.9 |
| *Rhododendron canadense* | 32.7 | 37.0 | 2.42 | 191.0 | 0.345 | 1.98 | 25.7 |
| *Rhododendron groenlandicum* | 20.5 | 55.7 | 1.09 | 112.1 | 0.448 | 1.93 | 34.1 |
| *Roripa amphibia* | 47.3 | 38.0 | 37.40 | 393.6 | 0.113 | 0.55 | 17.0 |
| *Rubus chamaemorus* | 39 | 60.8 | 26.60 | 167.2 | 0.404 | 1.96 | 24.8 |
| *Rynchospora alba* | 22.9 | 15.6 | 1.22 | 123.2 | 0.430 | 1.43 | 46.2 |
| *Sagittaria latifolia* | 54.4 | 42.6 | 71.70 | 329.4 | 0.167 | 1.30 | 27.2 |
| *Salix pedicellaris* | 31.9 | 23.5 | 3.02 | 149.6 | 0.392 | 1.74 | 33.5 |
| *Salix pyridifolia* | 38.9 | 37.8 | 7.71 | 162.2 | 0.431 | 1.83 | 23.9 |
| *Sarracenia purpurea* | 14.1 | 57.9 | 35.40 | 95.9 | 0.234 | 1.55 | 18.2 |
| *Schoenoplectus tabernaemontani* | 125 | 5.3 | 6.20 | 57.7 | 0.238 | 1.59 | 50.5 |
| *Scirpus pedicellatus* | 105 | 10.4 | 51.60 | 141.0 | 0.322 | 1.21 | 27.8 |
| *Scutellaria lateriflora* | 90.1 | 58.0 | 10.50 | 644.0 | 0.194 | 1.17 | 20.4 |
| *Sparganium eurycarpum* | 101 | 12.8 | 86.30 | 171.1 | 0.191 | 1.06 | 41.6 |
| *Spiraea latifolia* | 34 | 48.8 | 3.18 | 197.3 | 0.471 | 1.95 | 20.7 |
| *Spiraea tomentosa* | 39.1 | 43.8 | 3.24 | 209.1 | 0.449 | 1.98 | 25.3 |
| *Thelypteris palustris* | 45.6 | 71.8 | 54.00 | 342.0 | 0.175 | 0.94 | 16.0 |
| *Typha latifolia* | 166 | 7.5 | 102.00 | 99.1 | 0.231 | 1.35 | 51.0 |
| *Vaccinium angustifolium* | 16.4 | 29.7 | 2.01 | 157.8 | 0.341 | 1.90 | 33.7 |
| *Vaccinium macrocarpon* | 24.2 | 50.5 | 0.49 | 125.4 | 0.412 | 1.92 | 41.5 |
| *Vaccinium oxycoccos* | 18.8 | 67.0 | 0.12 | 90.8 | 0.445 | NA | NA |

1. Bayesian hierarchical distributional modelling
   1. Framework

The classical framework of the generalized linear models (GLM) allows modeling any random variable distributed following an exponential family distribution (such as normal, poisson, gamma…). Many of these distributions are constantly bounded, implying a nonlinear variation of their mean and variance close to these bounds. For example, the variation of the mean of a strictly positive value, such as a count, is not linear close to zero, just as the variation of a probability is nonlinear close to its bounds (zero and one). The GLM framework allows to model the effects of linear predictors on a *function* of the response variable, which makes the latter linear. The corresponding function is caller the *link function*, $g()$. The variance, which has also a non-linear behavior at the bound, is then linked to the mean by a *variance function* (Smyth 1989). Thus, the variation of the mean and variance of an exponential family distribution following a series of linear predictors $\mathbf{x}_{\mathbf{i}}^{\mathbf{T}}\boldsymbol{\beta}$ is

$$\begin{matrix} \mu_{i}=g^{-1}(\mathbf{x}_{\mathbf{i}}^{\mathbf{T}}\boldsymbol{\beta}) \\ \sigma_{i}^{2}=\phi_{i}w_{i}^{-1}v(\mu_{i}) \end{matrix}$$

Where $\mu_{i}$ and $\sigma_{i}^{2}$ are the mean and the variance of the distribution for the observation $i$, respectively, and $g^{-1}$ the inverse of the link function. $v()$ is a non-negative variance function specific to each probability distribution and $w_{i}^{-1}$ are known weights. $\phi_{i}$ is an unknown dispersion parameter for the observation $i$.

- 1. Distributional modelling

Many distributions, even if they are not properly belonging to the exponential family, might be reparametrized to model conjointly mean and variance. Given the fact that we can define the link between mean and variance as below, one would be able to regress both $\mu_{i}$ and $\phi_{i}$ as the result of deterministic equations. In this framework, we can define a distributional model for location and scale as follows (Rigby & Stasinopoulos 2005):

$$\begin{matrix} \mathbf{Y}\sim f(\boldsymbol{\mu},\boldsymbol{\phi}) \\ g_{1}(\boldsymbol{\mu})=\mathbf{X}\beta\\ g_{2}(\boldsymbol{\phi})=\mathbf{X}\gamma\end{matrix}$$

Where $\mathbf{Y}$ is a column vector of the response variable, $\boldsymbol{\mu}$ and $\boldsymbol{\phi}$ are vectors of linear parameters, $\mathbf{X}$ a matrix of predictors, and $\beta$ and $\gamma$ are vector coefficients linking predictors to the mean and the dispersion, respectively. $g_{1}()$ and $g_{2}()$ are the link function for the mean and dispersion, respectively.

- - 1. Gamma distribution

We modeled strictly positive vegetative height with a gamma distribution, with $\boldsymbol{\alpha}$ being the shape parameter and $\boldsymbol{\beta}$ being the rate parameter. The formulation with independent mean, $\boldsymbol{\mu}$ and dispersion, $\boldsymbol{\phi}$, is as follows:

$$\begin{matrix} \mathbf{Y}\sim Gamma(\boldsymbol{\alpha}=\boldsymbol{\mu}^{2}\boldsymbol{\phi},\boldsymbol{\beta}=\boldsymbol{\mu\phi}) \\ log(\boldsymbol{\mu})=\mathbf{X}\beta\\ log(\boldsymbol{\phi})=\mathbf{X}\gamma\end{matrix}$$

This parametrization is straightforward to recover, given that the first two moments of the gamma distribution are defined as follows:

$$\begin{matrix} E(Y)=\frac{\alpha}{\beta} \\ Var(Y)=\frac{\alpha}{\beta^{2}} \end{matrix}$$

- - 1. Beta distribution

Ferrari and Cribari-Neto (2004) proposed a formulation of the beta regression for modelling rates or proportion. In this framework, the two shape parameters of the beta distribution might be reformulated dependently of a mean, $\mu$ and a precision parameter, $\phi$.

$$\begin{matrix} \mathbf{Y}\sim Beta(\mathbf{p}=\boldsymbol{\mu\phi},\mathbf{q}=(1-\boldsymbol{\mu})\boldsymbol{\phi}) \\ logit(\boldsymbol{\mu})=\mathbf{X}\beta\\ log(\boldsymbol{\phi})=\mathbf{X}\gamma\end{matrix}$$

$\phi$ is a precision parameter given the fact that, for a fixed $\mu$, the greater it is, the lower is the variance. The relationship between the precision and the variance is as follow:

$$Var(Y)=\frac{V(\mu)}{1+\phi}=\frac{\mu(1-\mu)}{1+\phi}$$

$V()$ is a variance function. While variance and mean are not completely independent, it is straightforward to compare a model with fixed precision to a model with precision determined by a deterministic equation, because the variance is similarly influenced by the variation of the mean in both cases.

- 1. Hierarchical model formulation

Because of the structured nature of our design (individuals within species, within communities, within ecosystems), we used hierarchical parameters for slopes and intercepts within ecosystems. Thus, intercepts for each community within an ecosystem where modeled as belonging to the same population with estimated variance. The complete formulation of the model $M_{com3}$, which explore the relationship between species diversity and mean and dispersion within each ecosystem, is as follows:

$$\begin{matrix} y_{i}\sim f(\mu_{i},\phi_{i}) \\ g_{1}(\mu_{i})=\beta_{0}+\beta_{T2}+\beta_{Ee}+\beta_{Cc}+\beta_{1Ee}D_{p} \\ log(\phi_{i})=\gamma_{0}+\gamma_{T2}+\gamma_{Ee}+\gamma_{Cc}+\gamma_{1Ee}D_{p} \\ \beta_{P}\sim normal(0,\sigma_{\beta_{C}}) \\ \gamma_{P}\sim normal(0,\sigma_{\gamma_{C}}) \end{matrix}$$

Where $y_{i}$ is the trait value of individual $i$. The $\beta$ and $\gamma$ parameters are the coefficients for mean and dispersion, respectively. Thus, $\beta_{0}$ and $\gamma_{0}$ are the intercepts, while $\beta_{T2}$ and $\gamma_{T2}$ are the coefficient for the individuals harvested during the second sampling campaign. $\beta_{Ee}$ and $\gamma_{Ee}$ are deviation parameters for each ecosystem *e*, and $\beta_{Cc}$ and $\gamma_{Cc}$ are hierarchical intercepts for each plot within ecosystem p. $\beta_{1Ee}$ and $\gamma_{1Ee}$ are the slopes linking taxonomic diversity to mean and dispersion within each ecosystem. We used weakly informative priors to optimize posterior sampling with appropriate constraints and scaling, without eliciting actual knowledge, which are defined as follows, with *hstudent* being a truncated student-t distribution defined over positive values:

$$\begin{matrix} \beta_{0}\sim student(3,x,10) \\ \beta_{T2}\sim student(3,0,5) \\ \beta_{Ee}\sim student(3,0,5) \\ \beta_{1Ee}\sim student(3,0,5) \\ \sigma_{\beta_{Cc}}\sim hstudent(3,0,5) \end{matrix}\begin{matrix} \gamma_{0}\sim student(3,0,10) \\ \gamma\sim student(3,0,5) \\ \gamma\sim student(3,0,5) \\ \gamma_{1E}\sim student(3,0,5) \\ \sigma_{\gamma_{P}}\sim hstudent(3,0,5) \end{matrix}$$

With *x* a scalar automatically scaled by the *brms* package to represent the data.

When modelling the response of species trait distribution to the diversity gradient, we modeled both species intercepts and slopes as distributed multinormally with estimated covariance matrix. The complete formulation of the model $M_{sp3}$is as follows:

$$\begin{matrix} y_{i}\sim f(\mu_{i},\phi_{i}) \\ g_{1}(\mu_{i})=\beta_{0}+\beta_{T2}+\beta_{Ee}+\beta_{Cc}++\beta_{Ss}+\beta_{1Ss}{yD}_{p} \\ log(\phi_{i})=\gamma_{0}+\gamma_{T2}+\gamma_{Ee}+\gamma_{Cc}+\gamma_{Ss}+\gamma_{1Ss}D_{p} \\ \beta_{Cc}\sim normal(0,\sigma_{\beta_{Cc}}) \\ \gamma_{Cc}\sim normal(0,\sigma_{\gamma_{Cc}}) \\ \left[ \begin{matrix} \beta_{Ss} \\ \beta_{1Ss} \\ \gamma_{Ss} \\ \gamma_{1Ss} \end{matrix} \right]=MVNormal(\left[ \begin{matrix} 0 \\ 0 \\ 0 \\ 0 \end{matrix} \right],S) \end{matrix}$$

S being a covariance matrix computed as follow:

$$\begin{matrix} S=\left[ \begin{matrix} \sigma_{\beta_{Ss}} & 0 & 0 & 0 \\ 0 & \sigma_{\beta_{1Ss}} & 0 & 0 \\ 0 & 0 & \sigma_{\gamma_{Ss}} & 0 \\ 0 & 0 & 0 & \sigma_{\gamma_{1Ss}} \end{matrix} \right]R\left[ \begin{matrix} \sigma_{\beta_{Ss}} & 0 & 0 & 0 \\ 0 & \sigma_{\beta_{1Ss}} & 0 & 0 \\ 0 & 0 & \sigma_{\gamma_{Ss}} & 0 \\ 0 & 0 & 0 & \sigma_{\gamma_{1Ss}} \end{matrix} \right] \end{matrix}$$

Where $R$ is an estimated 4x4 correlation matrix. The priors for this model are follow, the LKJcorr distribution being a modified beta distribution to be a prior of correlation matrices.

$$\begin{matrix} \beta_{0}\sim student(3,x,10) \\ \beta_{T2}\sim student(3,0,5) \\ \beta_{Ee}\sim student(3,0,5) \\ \sigma_{\beta_{Cc}}\sim hstudent(3,0,5) \\ \sigma_{\beta_{Ss}}\sim hstudent(3,0,5) \\ \sigma_{\beta_{1Ss}}\sim hstudent(3,0,5) \end{matrix}\begin{matrix} \gamma_{0}\sim student(3,0,10) \\ \gamma_{T2}\sim student(3,0,5) \\ \gamma_{Ee}\sim student(3,0,5) \\ \sigma_{\gamma_{Cc}}\sim hstudent(3,0,5) \\ \sigma_{\gamma_{Ss}}\sim hstudent(3,0,5) \\ \sigma_{\gamma_{1Ss}}\sim hstudent(3,0,5) \end{matrix}\begin{matrix} R\sim LKJcorr(2) \end{matrix}$$

- 1. Model diagnosis

*Sampling behaviors* - The *No-U-Turn sampler* implemented in stan returned several diagnostics. For every model, we ensured that there were no divergent transitions. Divergent transitions arise when the curvature of an area of the posterior surface is too high to be adequately explored by the sampler. This diagnostic is very important, and this situation shall be avoided, because it leads to biased estimates (Gelman *et al.* 2013). Well-chosen priors and the parametrized Stan language produced by *brms* (Bürkner 2018) avoided such suboptimal sampling behavior, and parametrizing the step size at 0.95 in the sampler was sufficient to fit every model without divergences. Once the sampler behaves correctly, the second important concern is the mixing of the different chains. The chains represent independent instances of posterior surface exploration, beginning at various starting points. A rule of thumb is that a Gelman-Rubin split $\overset{^}{R}$ greater than 1.1 indicates bad mixing of chains (Gelman *et al.* 2013). Every $\overset{^}{R}$ in the models were lower than 1.01. We also inspected each chain visually to ensure the absence of problematic behaviors. Each chain were constituted of 2000 iterations including 1000 warm-up iterations.

*Posterior-predictive checks* - We checked that models recovered key features of data by confronting visually predicted and observed distributions at each biological organization scale (ecosystem, community, species; see Figs. S20-S47).

- 1. Model predictions

We operated predictions at ecosystem, community and species levels.

- - 1. Ecosystem level predictions


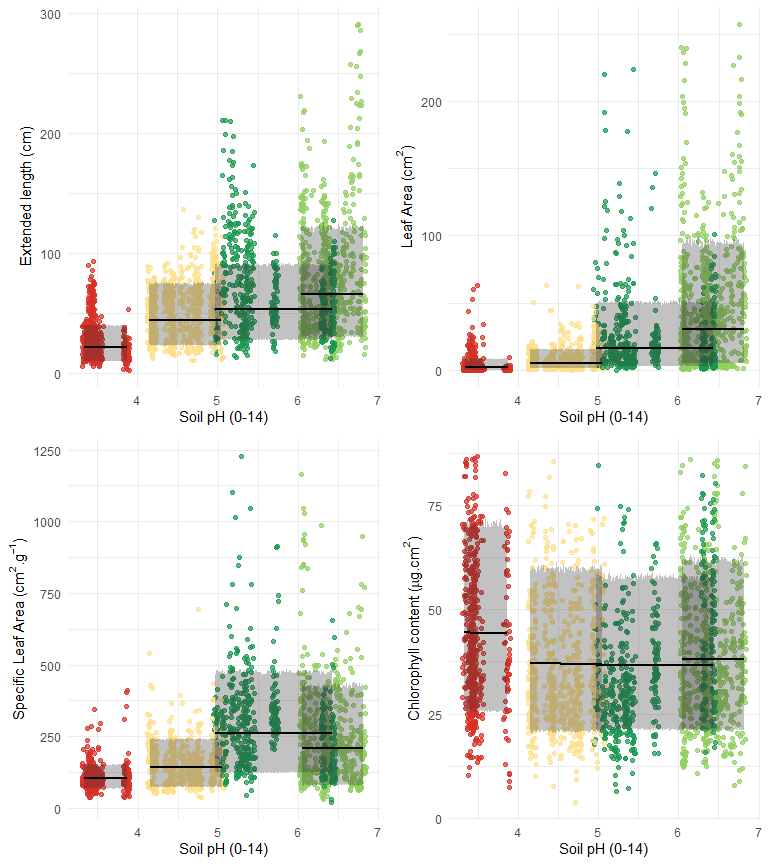


**Figure S4**: Between ecosystem differences for the seven studied functional traits. Lines represent the median predicted value, and the shaded areas represent the 80% predictive interval. Red: bog; Yellow: fen; Light green: wet meadow; Dark green: fluvial marsh.


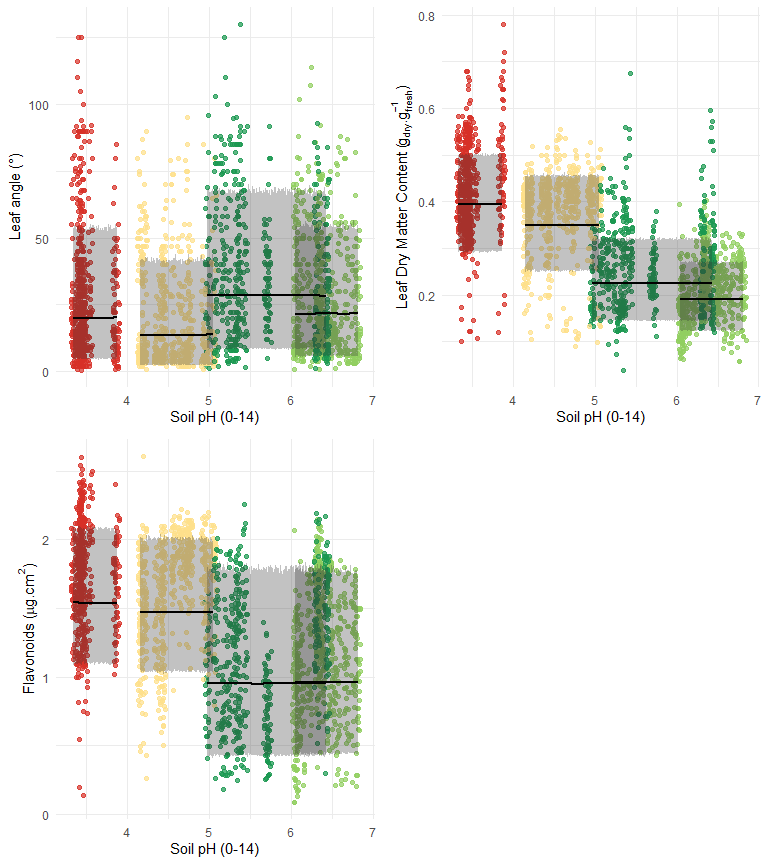


**Figure S5**: Between ecosystem differences for the seven studied functional traits. Lines represent the median predicted value, and the shaded areas represent the 80% predictive interval. Red: bog; Yellow: fen; Light green: wet meadow; Dark green: fluvial marsh.

- - 1. Community level predictions

**Table S2:** Summary of estimated communities’ trait distribution in response to plant species richness. We displayed 90% credible intervals of exponentiated slopes (parameter β_1E_ for *µ* and parameter γ_1E_ for σ and *ϕ* in Eqs. 2). *µ* represents the mean of the distribution of trait values, while σ represents its dispersion (for gamma distribution) and *ϕ* its precision (for the particular beta distribution of LDMC). Value intervals that characterize negative slopes are in blue font (when *µ* and σ > 1, and *ϕ* <1), ones that characterize positive ones are in red font (opposite trends), while ones that include 1 do not show directional trend. Overall represents the global mean response across the data set. Predictions are available in Fig. S5-S11. Abbreviations: Angle: leaf angle; Chlo.: leaf chlorophyll content; EL: extended length; Flav.: leaf flavonoid content; LA: leaf area; LDMC: leaf dry matter content; SLA: specific leaf area.

| **Ecosystem** | **Param.** | **Angle** | **EL** | **LA** | **SLA** | **Chlo.** | **LDMC** | **Flav.** |
| --- | --- | --- | --- | --- | --- | --- | --- | --- |
| Overall | µ | **1.02-1.08** | **1.03-1.08** | 0.99-1.03 | **1.01-1.04** | **0.95-0.98** | 0.99-1.02 | 0.96-1.01 |
|  | σ *or ϕ* | **1.02-1.20** | 0.96-1.13 | **1.07-1.18** | **1.18-1.35** | **1.02-1.16** | **0.88-0.93** | **1.08-1.20** |
| Bog | µ | **1.03-1.16** | **1.06-1.17** | 0.98-1.05 | **1.01-1.06** | **0.90-0.96** | 0.99-1.03 | 0.99-1.07 |
|  | σ *or ϕ* | **1.04-1.43** | 0.93-1.29 | **1.14-1.39** | **1.38-1.82** | **1.03-1.34** | **0.78-0.86** | **1.16-1.44** |
| Fen | µ | **1.01-1.06** | 0.98-1.01 | **1.05-1.15** | **1.01-1.04** | **0.97-0.99** | 0.99-1.03 | 0.99.1.01 |
|  | σ *or ϕ* | **1.01-1.14** | 0.94-1.03 | **1.21-1.53** | **1.04-1.14** | 0.94-1.02 | **0.76-0.86** | 0.94-1.02 |
| Meadow | µ | 0.98-1.02 | **1.08-1.20** | 0.99-1.04 | 0.98-1.02 | **0.95-0.98** | 0.99-1.02 | **0.97-0.99** |
|  | σ *or ϕ* | **1.01-1.12** | **1.13-1.30** | **1.01-1.15** | **1.10-1.24** | 0.96-1.05 | **0.93-0.99** | **1.09-1.19** |
| Marsh | µ | **1.01-1.19** | **1.07-1.12** | **0.90-0.98** | **1.01-1.06** | **0.85-0.98** | 0.97-1.01 | 0.97-1.01 |
|  | σ *or ϕ* | 0.81-1.49 | **1.05-1.11** | 0.88-1.09 | **1.33-1.38** | **1.02-1.75** | **0.89-0.98** | **1.12-1.78** |

**Figure S6**: Relationship between species diversity and extended length community trait distribution in every ecosystem. Lines represent the median predicted value, and the shaded areas represent the 80% predictive interval. Red: bog; Yellow: fen; Light green: Fluvial marsh; Dark green: wet meadow.


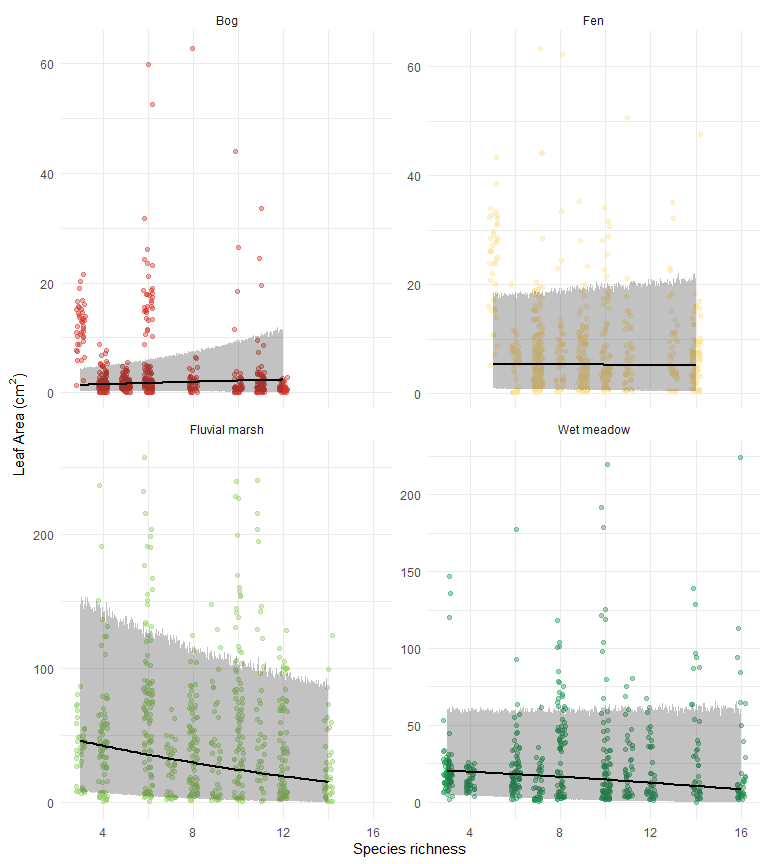


**Figure S7**: Relationship between species diversity and leaf area community trait distribution in every ecosystem. Lines represent the median predicted value, and the shaded areas represent the 80% predictive interval. Red: bog; Yellow: fen; Light green: Fluvial marsh; Dark green: wet meadow.


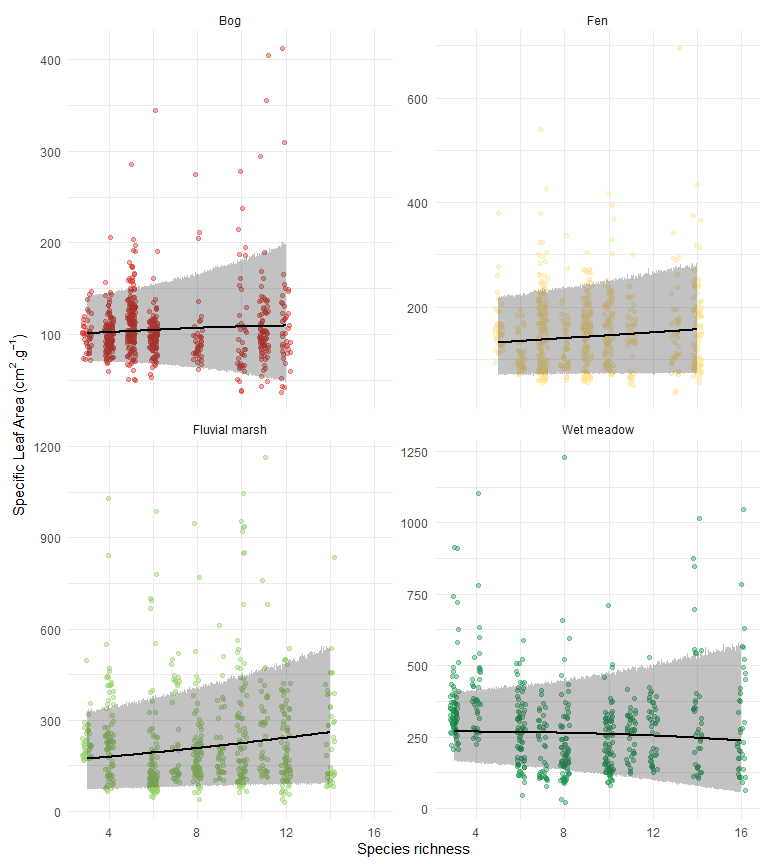


**Figure S8**: Relationship between species diversity and specific leaf area community trait distribution in every ecosystem. Lines represent the median predicted value, and the shaded areas represent the 80% predictive interval. Red: bog; Yellow: fen; Light green: Fluvial marsh; Dark green: wet meadow.


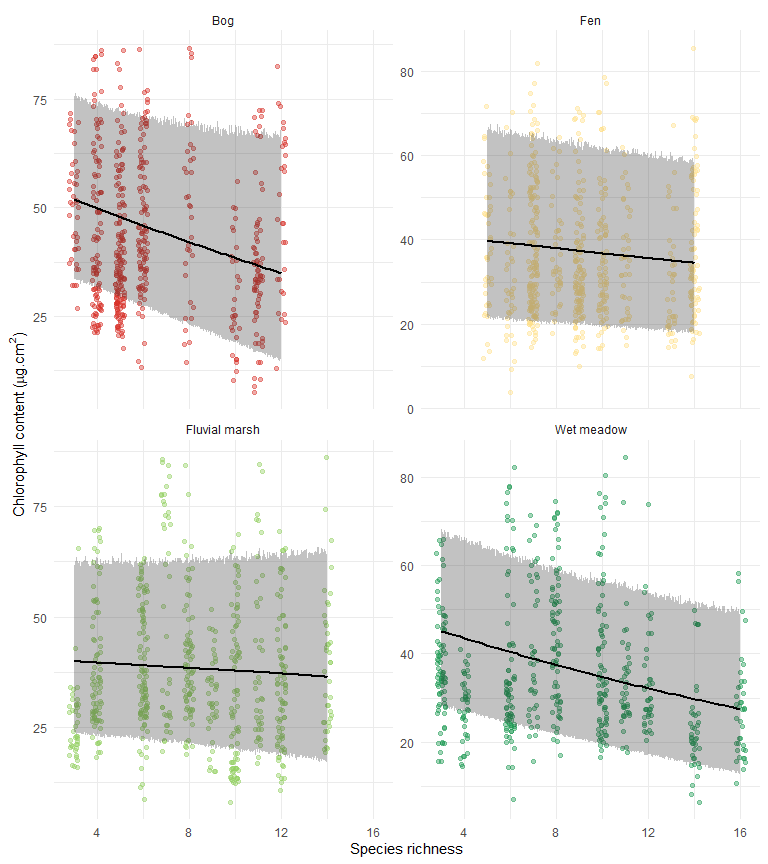


**Figure S9**: Relationship between species diversity and superficial chlorophyll community trait distribution in every ecosystem. Lines represent the median predicted value, and the shaded areas represent the 80% predictive interval. Red: bog; Yellow: fen; Light green: Fluvial marsh; Dark green: wet meadow.


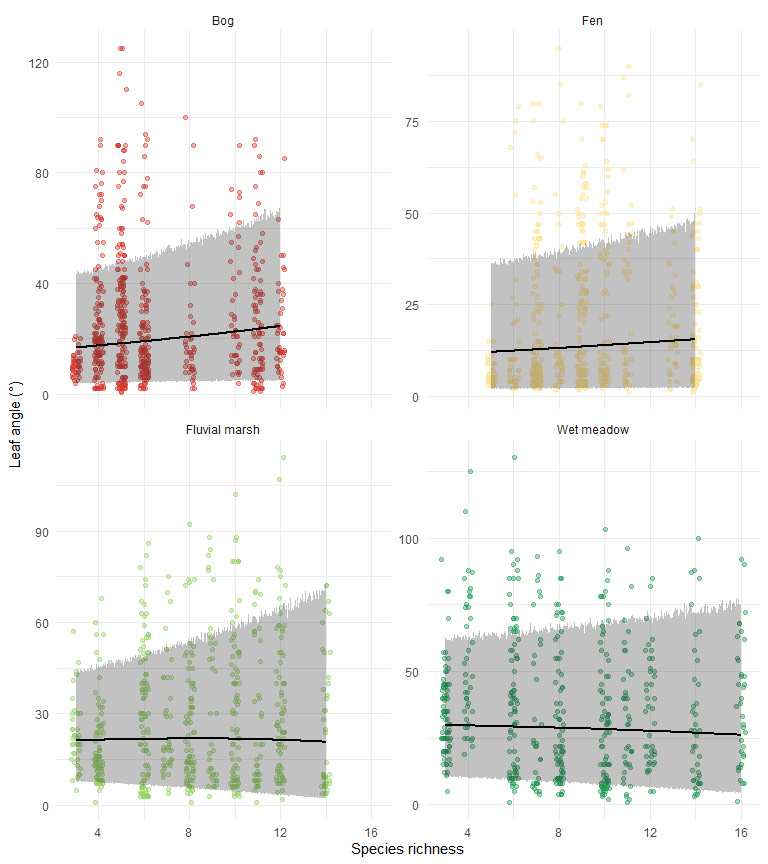


**Figure S10**: Relationship between species diversity and leaf angle community trait distribution in every ecosystem. Lines represent the median predicted value, and the shaded areas represent the 80% predictive interval. Red: bog; Yellow: fen; Light green: Fluvial marsh; Dark green: wet meadow.


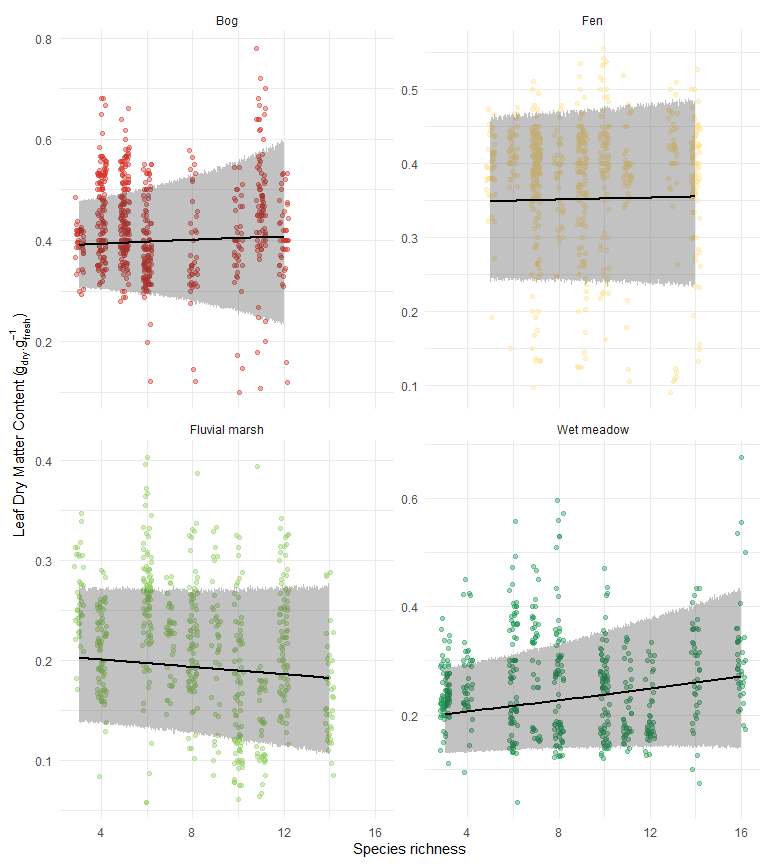


**Figure S11**: Relationship between species diversity and leaf dry matter content community trait distribution in every ecosystem. Lines represent the median predicted value, and the shaded areas represent the 80% predictive interval. Red: bog; Yellow: fen; Light green: Fluvial marsh; Dark green: wet meadow.


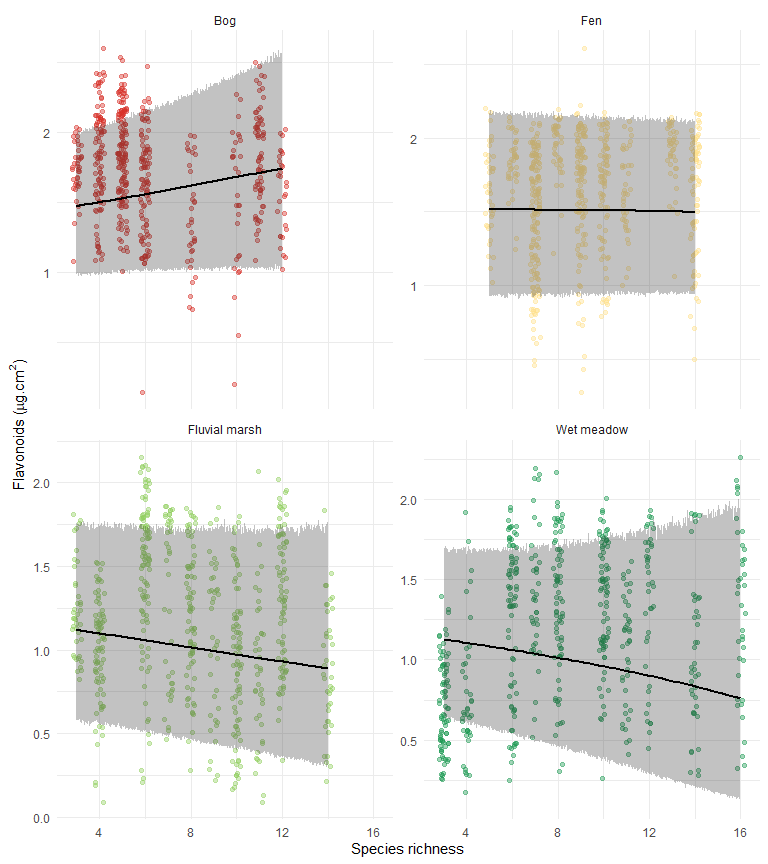


**Figure S12**: Relationship between species diversity and flavonoids community trait distribution in every ecosystem. Lines represent the median predicted value, and the shaded areas represent the 80% predictive interval. Red: bog; Yellow: fen; Light green: Fluvial marsh; Dark green: wet meadow.

- - 2. Species level predictions

**Table S3**: Summary of estimated species slopes in the relationships of plant functional and demographic traits with plant species richness.

| **Ecosyst.** | **Species** | **Density** | **Param.** | **Angle** | **EL** | **LA** | **SLA** | **Chlo.** | **LDMC** | **Flavo.** |
| --- | --- | --- | --- | --- | --- | --- | --- | --- | --- | --- |
| Bog | *Chamaedaphne calyculata* | -0.03  (-0.07 0) | mu | 1.00  (0.99-1.01) | 1.00  (0.99-1.01) | 1.02  (1- 1.04) | 1.00  (0.98-1.00) | 1.00  (1.00 1.01) | 1.00  (1.00 1.01) | 1.00  (1.00 1.01) |
| Bog | *Chamaedaphne calyculata* | NA | phi | 1.00  (0.94-1.05) | 1.10  (1.03 1.18) | 1.09  (1.02-1.18) | 0.98  (0.92-1.05) | 0.99  (0.94 1.04) | 0.97  (0.91 1.01) | 1.00  (0.96 1.02) |
| Bog | *Eriophorum virginicum* | -0.08  (-0.12 -0.04) | mu | 1.00  (0.99-1.01) | 1.00  (0.99 1.01) | 1.00  (0.98-1.02) | 1.02  (1.01-1.03) | 1.00  (0.99 1.01) | 1.00  (0.99 1.00) | 1.00  (0.99 1.00) |
| Bog | *Eriophorum virginicum* | NA | phi | 1.03  (0.98-1.11) | 0.99  (0.92 1.07) | 1.00  (0.94-1.07) | 1.04  (1.01 1.09) | 1.06  (1.00 1.15) | 0.97  (0.91 1.02) | 1.01  (0.98 1.05) |
| Fen | *Carex lasiocarpa* | 0.05  (0.02 0.09) | mu | 1.00  (0.99-1.01) | 1.00  (1.00 1.01) | 1.01  (1-1.03) | 1.00  (0.99-1.00) | 1.00  (1.00 1.01) | 1.00  (0.99 1.00) | 1.00  (1.00 1.01) |
| Fen | *Carex lasiocarpa* | NA | phi | 0.96  (0.91-1.01) | 0.89  (0.84 0.96) | 1.01  (0.96-1.07) | 0.94  (0.88-1.00) | 0.96  (0.90 1.01) | 1.02  (0.98 1.07) | 1.00  (0.97 1.02) |
| Fen | *Carex oligosperma* | -0.26  (-0.3 -0.22) | mu | 1.00  (0.99-1.02) | 1.00  (0.99 1.00) | 1.01  (0.99-1.03) | 1.00  (1 1.02) | 1.00  (0.99 1.01) | 1.00  (0.99 1.00) | 1.00  (0.99 1.00) |
| Fen | *Carex oligosperma* | NA | phi | 1.08  (1.02-1.16) | 1.01  (0.93 1.09) | 1.07  (1.01-1.15) | 1.18  (1.09 1.28) | 0.94  (0.88 1.00) | 0.93  (0.86 0.99) | 1.00  (0.96 1.03) |
| Fen | *Carex sect. Phacocystis* | -0.18  (-0.22 -0.14) | mu | 1.01  (1.00-1.03) | 1.00  (0.99 1.00) | 0.97  (0.95-0.99) | 1.01  (1.00 1.02) | 1.00  (0.98 1.00) | 1.00  (1.00 1.01) | 1.00  (0.99 1.01) |
| Fen | *Carex sect. Phacocystis* | NA | phi | 1.06  (1.00-1.14) | 0.98  (0.91 1.05) | 0.98  (0.92-1.04) | 1.07  (1.01 1.16) | 0.93  (0.86 1.00) | 0.96  (0.90 1.00) | 0.99  (0.96 1.02) |
| Marsh | *Acorus calamus* | -0.14  (-0.19 -0.08) | mu | 1.00  (0.99-1.02) | 1.00  (0.98 1.00) | 0.99  (0.97 1.02) | 1.00  (0.99 1.01) | 1.00  (0.99 1.01) | 1.00  (1.00 1.01) | 1.01  (1 1.01) |
| Marsh | *Acorus calamus* | NA | phi | 1.06  (1.00-1.15) | 1.35  (1.21 1.51) | 1.04  (0.98 1.13) | 1.00  (0.93 1.08) | 1.02  (0.96 1.09) | 0.96  (0.89 1.01) | 0.99  (0.96 1.02) |
| Marsh | *Comarum palustre* | 0.05  (-0.01 0.1) | mu | 1.00  (0.99-1.01) | 1.00  (0.99 1.01) | 1.03  (1.01 1.06) | 1.01  (1.00 1.03) | 1.00  (1.00 1.01) | 1.00  (1.00 1.01) | 1.00  (0.99 1.01) |
| Marsh | *Comarum palustre* | NA | phi | 0.99  (0.92-1.04) | 0.99  (0.91 1.07) | 0.99  (0.93 1.06) | 1.06  (0.99 1.16) | 0.93  (0.86 1.00) | 1.00  (0.95 1.05) | 1.01  (0.99 1.06) |
| Marsh | *Lythrum salicaria* | 0.03  (0 0.07) | mu | 1.00  (0.99-1.02) | 1.00  (0.99 1.01) | 0.98  (0.96 1.00) | 1.01  (1.00 1.02) | 1.00  (0.99 1.01) | 1.00  (0.99 1.00) | 1.00  (0.99 1.00) |
| Marsh | *Lythrum salicaria* | NA | phi | 1.03  (0.98 1.10) | 1.08  (1.00 1.15) | 1.06  (1.00 1.14) | 1.00  (0.94-1.06) | 0.96  (0.91 1.01) | 1.02  (0.98 1.08) | 1.01  (0.98 1.04) |
| Marsh | *Typha latifolia* | 0 (-0.04 0.03) | mu | 1.00  (0.98 1.01) | 1.00  (0.99 1.01) | 0.97  (0.95 1.00) | 1.00  (0.99-1.01) | 1.00  (0.99 1.00) | 1.00  (1.00 1.01) | 1.00  (0.99 1.00) |
| Marsh | *Typha latifolia* | NA | phi | 0.99  (0.93 1.04) | 0.99  (0.93 1.06) | 0.92  (0.84 0.99) | 0.95  (0.89 1.01) | 0.99  (0.94 1.04) | 1.01  (0.97 1.07) | 0.98  (0.93 1.01) |
| Meadow | *Acorus calamus* | -0.29  (-0.34 -0.23) | mu | 1.00  (0.99 1.02) | 1.00  (0.99 1.01) | 1.04  (1.01 1.08) | 1.00  (0.98 1) | 1.00  (0.99 1.01) | 1.00  (1.00 1.01) | 1.00  (1.00 1.01) |
| Meadow | *Acorus calamus* | NA | phi | 1.05  (0.99 1.14) | 1.25  (1.14 1.38) | 1.07  (0.99 1.18) | 0.96  (0.87 1.04) | 0.98  (0.91 1.05) | 0.95  (0.88 1.01) | 0.97  (0.91 1.01) |
| Meadow | *Lythrum salicaria* | 0.05  (0 0.11) | mu | 1.00  (0.98 1.01) | 1.00  (1.00 1.02) | 0.98  (0.96 1.00) | 1.00  (0.99 1.01) | 1.00  (0.99 1.01) | 1.00  (1.00 1.01) | 1.00  (0.99 1) |
| Meadow | *Lythrum salicaria* | NA | phi | 1.02  (0.96 1.11) | 0.98  (0.89 1.08) | 0.98  (0.91 1.04) | 1.04  (0.97 1.14) | 0.94  (0.85 1.01) | 1.02  (0.97 1.1) | 1.01  (0.99 1.06) |

Parameters were recovered from the best model for each trait, presented in table 1 (parameter β_1E_ for *µ* and parameter γ_1E_ for σ and *ϕ* in Eqs. 3). For sake of clarity, we resumed slopes by symbols representing their sign. Statistical values are presented in Table S We considered the inclusion of one in the 90% credible interval of parameters to attribute a sign. For the density, ‘decrease’ represented substantial variation of species mean relative density with species richness within each ecosystem, while ‘stable’ indicated no substantial variation. For each trait, symbols ‘+’ and ‘–’ represented substantial variation in mean trait value (µ) or dispersion (σ) in response to variation in community species richness. Model predictions are available in Fig. S12-18. See abbreviations in Table 1.


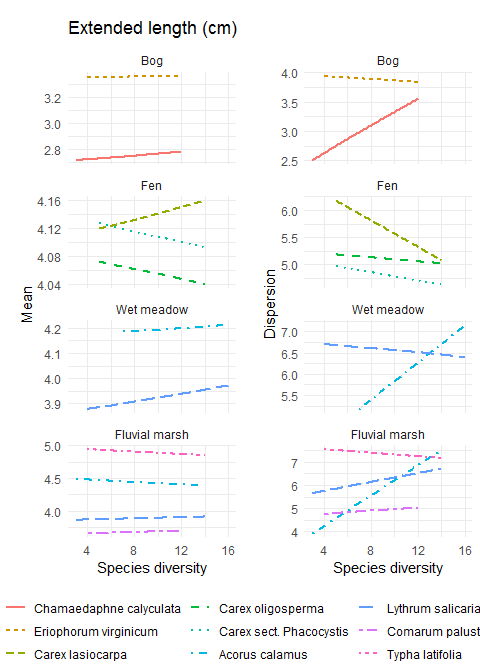


**Figure S13**: Relationship between species diversity and species extended length mean value and dispersion in every ecosystem. Lines represent the median predicted value of each parameter. Red: bog; yellow: fen; light green: wet meadow; dark green: fluvial marsh.


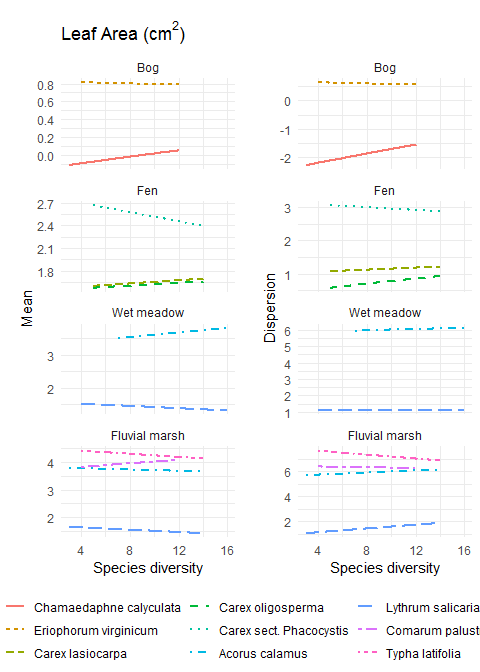


**Figure S14**: Relationship between species diversity and species leaf area mean value and dispersion in every ecosystem. Lines represent the median predicted value of each parameter. Red : bog; yellow : fen; light green: wet meadow; dark green: fluvial marsh.


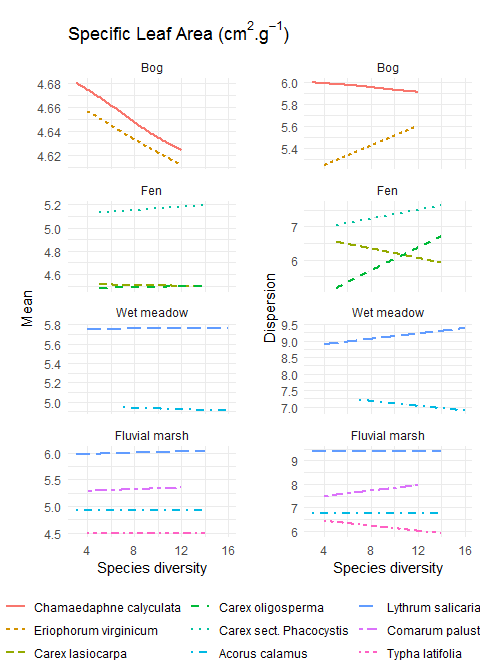


**Figure S15**: Relationship between species diversity and specific leaf area mean value and dispersion in every ecosystem. Lines represent the median predicted value of each parameter. Red: bog; yellow: fen; light green: wet meadow; dark green: fluvial marsh.


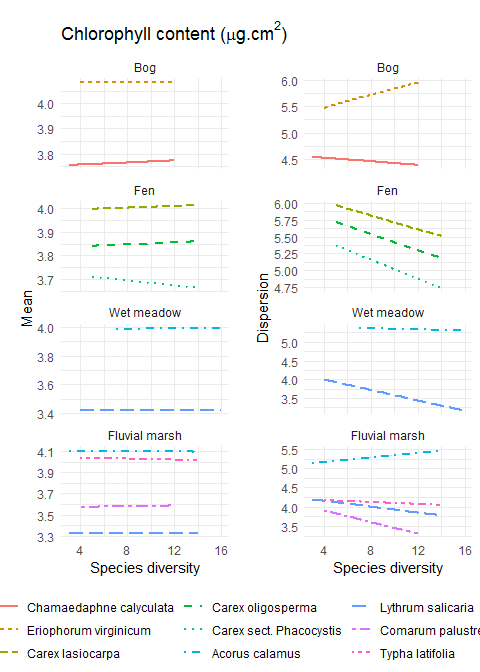


**Figure S16**: Relationship between species diversity and superficial chlorophyll mean value and dispersion in every ecosystem. Lines represent the median predicted value of each parameter. Red: bog; yellow: fen; light green: wet meadow; dark green: fluvial marsh.


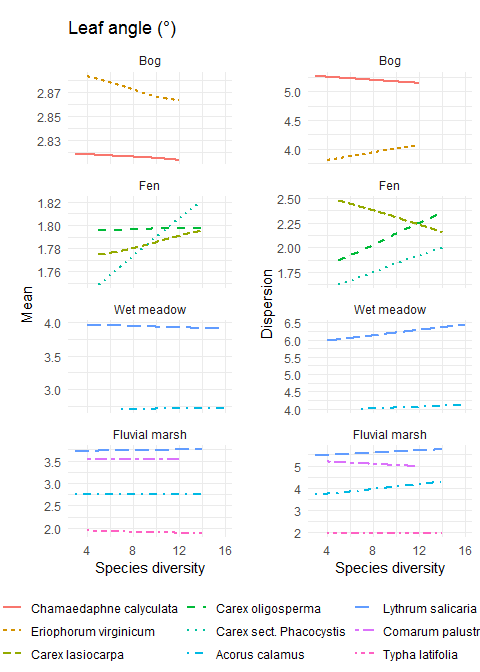


**Figure S17**: Relationship between species diversity and leaf angle mean value and dispersion in every ecosystem. Lines represent the median predicted value of each parameter. Red: bog; yellow: fen; light green: wet meadow; dark green: fluvial marsh.


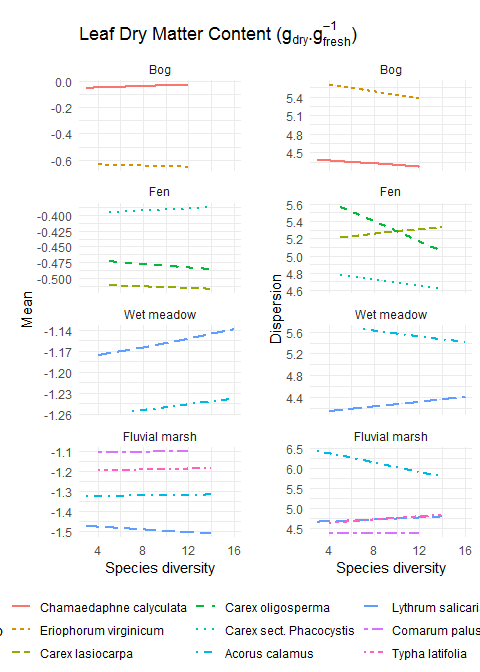


**Figure S18**: Relationship between species diversity and leaf dry matter content mean value and dispersion in every ecosystem. Lines represent the median predicted value of each parameter. Red: bog; yellow: fen; light green: wet meadow; dark green: fluvial marsh.


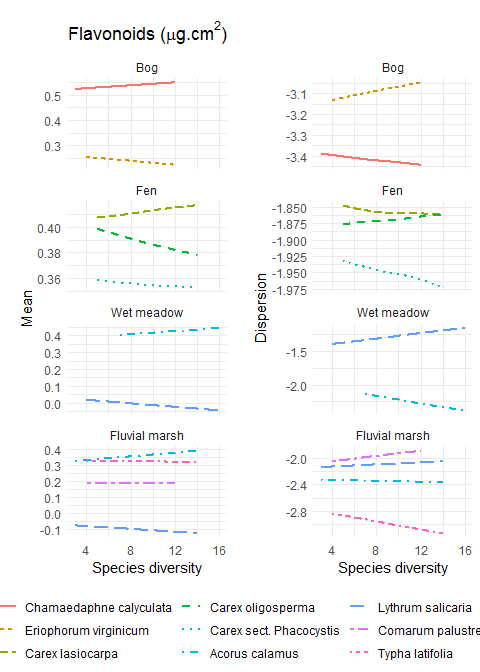


**Figure S19**: Relationship between species diversity and flavonoids mean value and dispersion in every ecosystem. Lines represent the median predicted value of each parameter. Red: bog; yellow: fen; light green: wet meadow; dark green: fluvial marsh.

- 1. Model diagnosis
     1. Community level


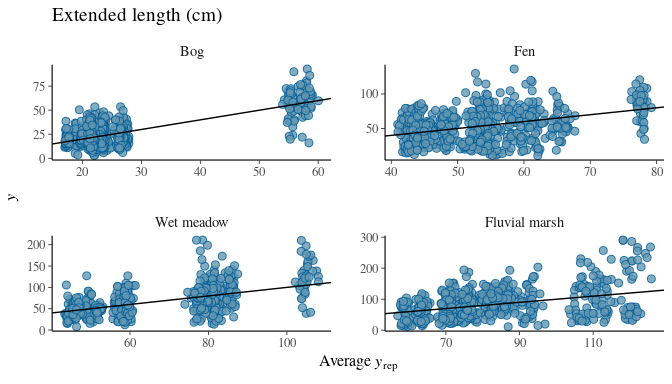


**Figure S20**: Fit between observed and predicted extended length values within each ecosystems. Line represent a 1:1 line.


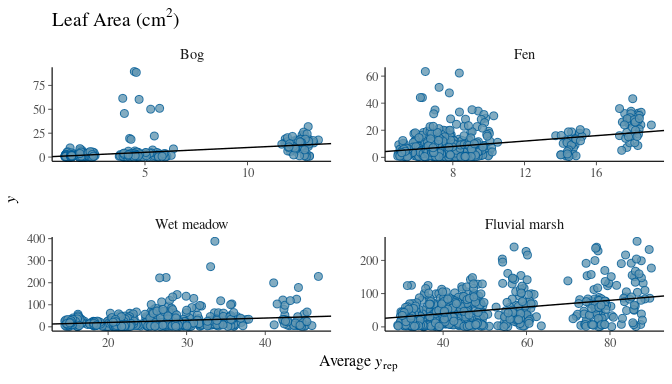


**Figure S21**: Fit between observed and predicted leaf area values within each ecosystems. Line represent a 1:1 line.


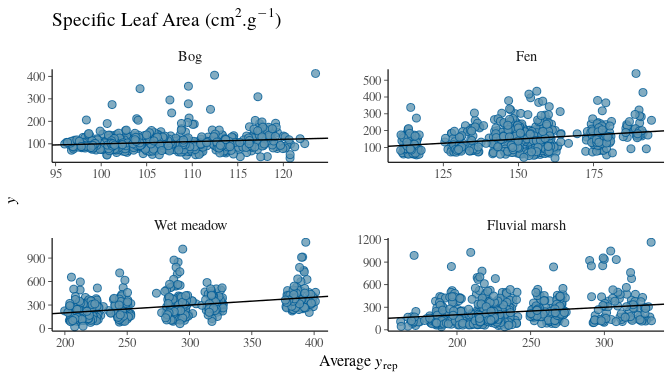


**Figure S22**: Fit between observed and predicted specific leaf area values within each ecosystems. Line represent a 1:1 line.


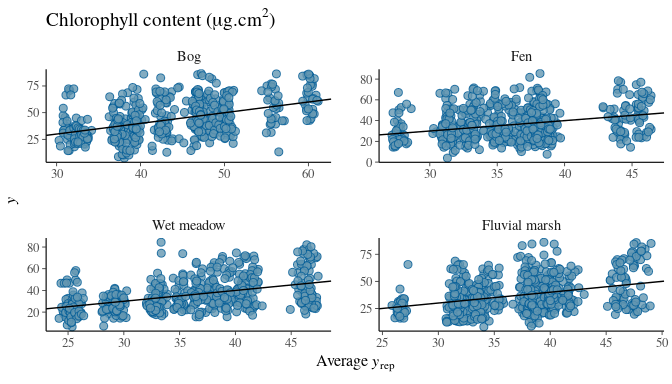


**Figure S23**: Fit between observed and predicted superficial chlorophyll values within each ecosystems. Line represent a 1:1 line.


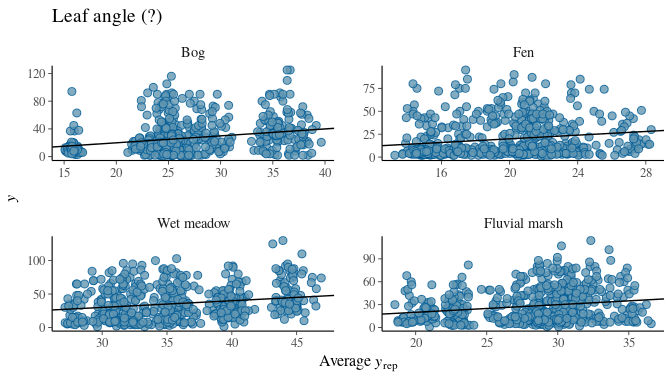


**Figure S24**: Fit between observed and predicted leaf angle values within each ecosystems. Line represent a 1:1 line.


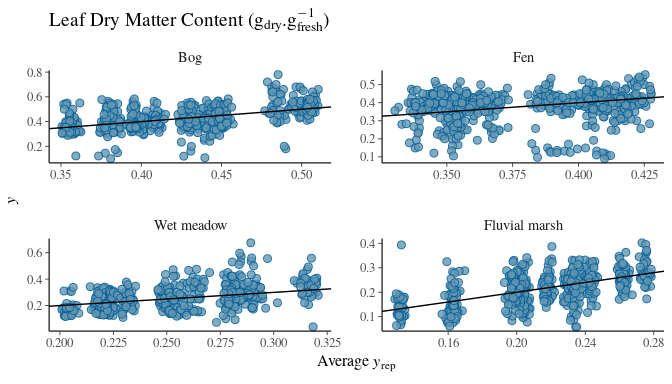


**Figure S25**: Fit between observed and predicted leaf dry matter content values within each ecosystems. Line represent a 1:1 line.


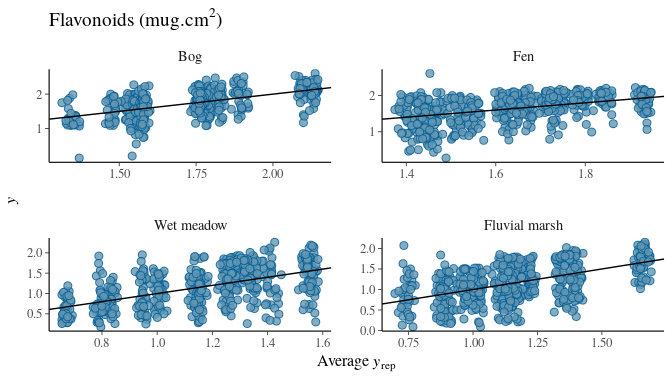


**Figure S26**: Fit between observed and predicted flavonoids values within each ecosystems. Line represent a 1:1 line.


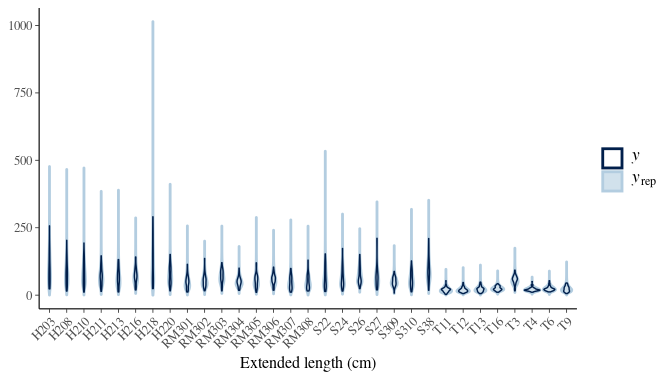


**Figure S27**: Violin plots representing the observed and predicted extended length distribution of each communities


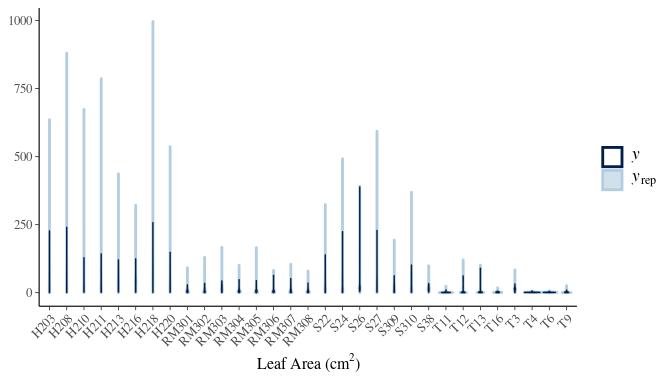


**Figure S28**: Violin plots representing the observed and predicted leaf area distribution of each communities


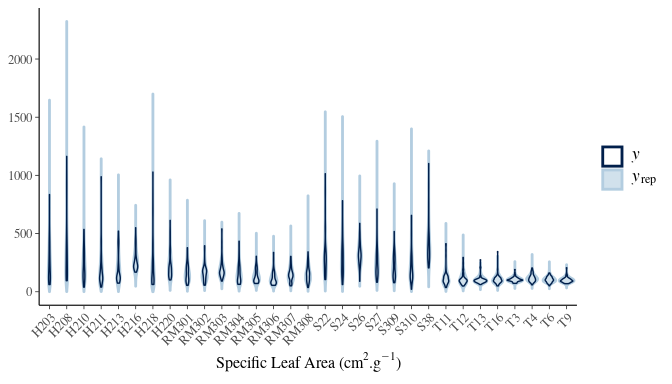


**Figure S29**: Violin plots representing the observed and predicted specific leaf area distribution of each communities


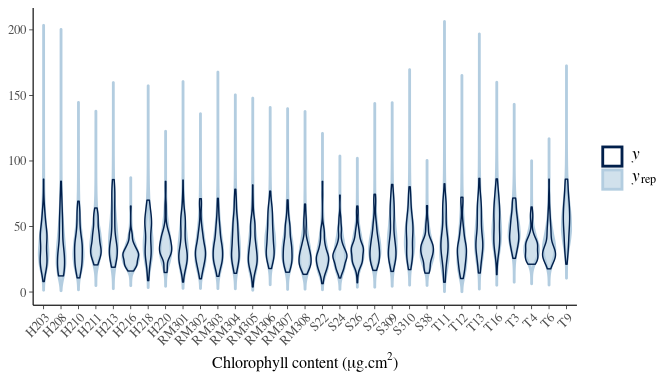


**Figure S30**: Violin plots representing the observed and predicted superficial chlorophyll distribution of each communities


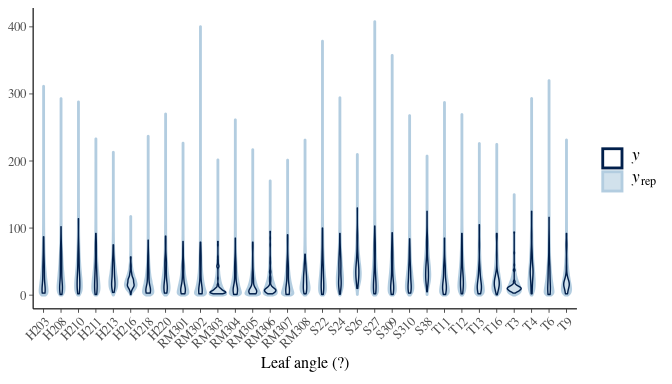


**Figure S31**: Violin plots representing the observed and predicted leaf angle distribution of each communities


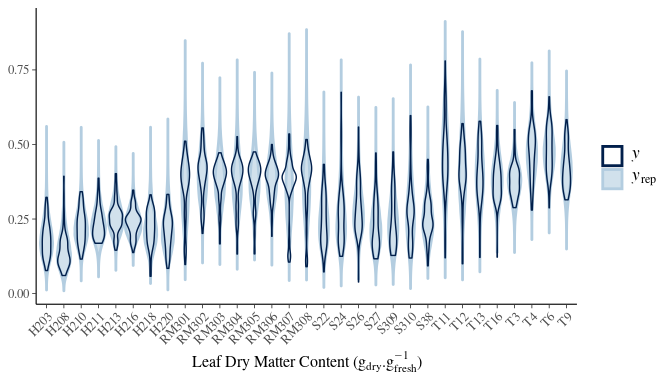


**Figure S32**: Violin plots representing the observed and predicted leaf dry matter content distribution of each communities


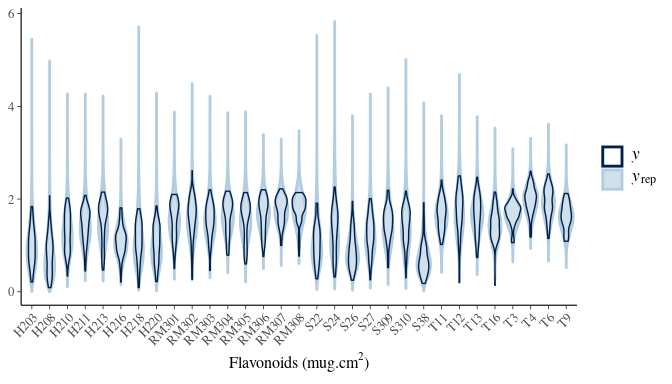


**Figure S33**: Violin plots representing the observed and predicted flavonoids distribution of each communities

- - 1. Species level


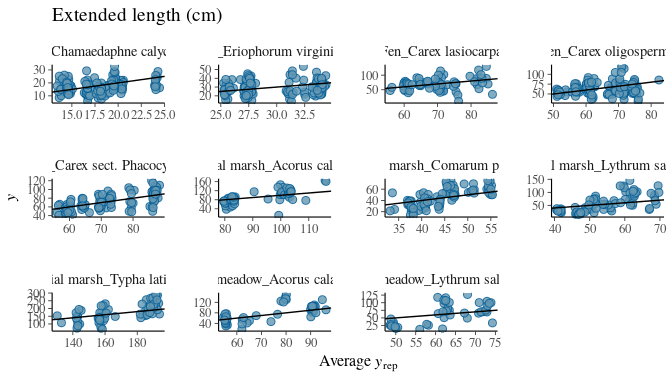


**Figure S34**: Fit between observed and predicted extended length values for each species in their ecosystem. Line represent a 1:1 line.


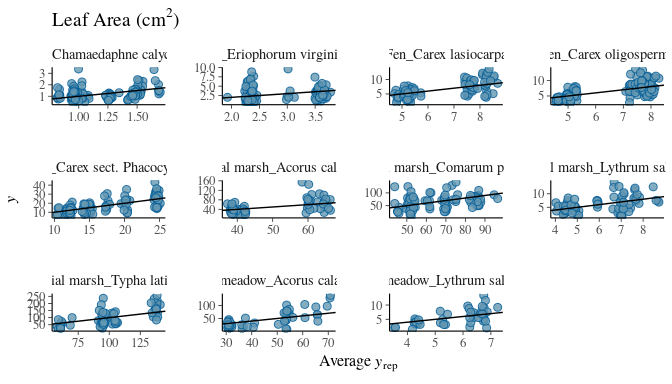


**Figure S35**: Fit between observed and predicted leaf area values for each species in their ecosystem. Line represent a 1:1 line.


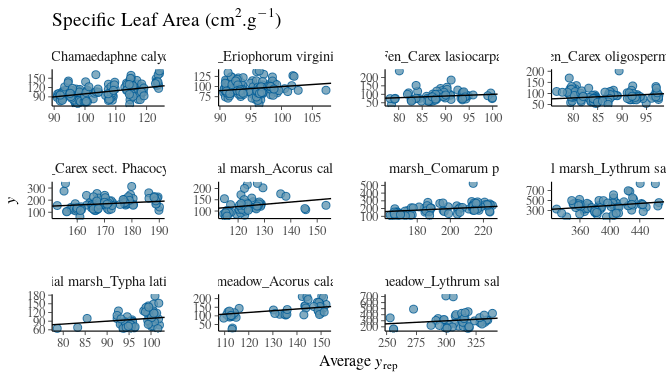


**Figure S36**: Fit between observed and predicted specific leaf area values for each species in their. Line represent a 1:1 line.


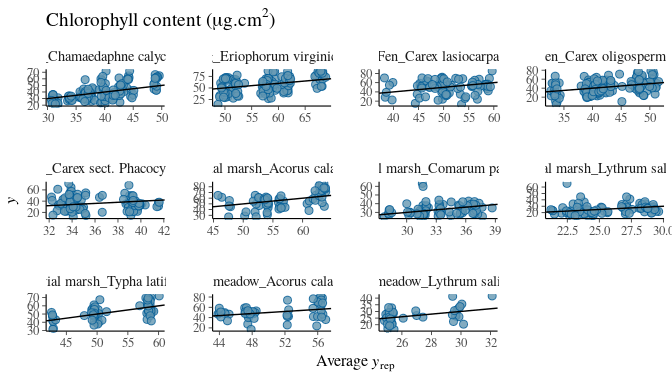


**Figure S37**: Fit between observed and predicted superficial chlorophyll values for each species in their. Line represent a 1:1 line.


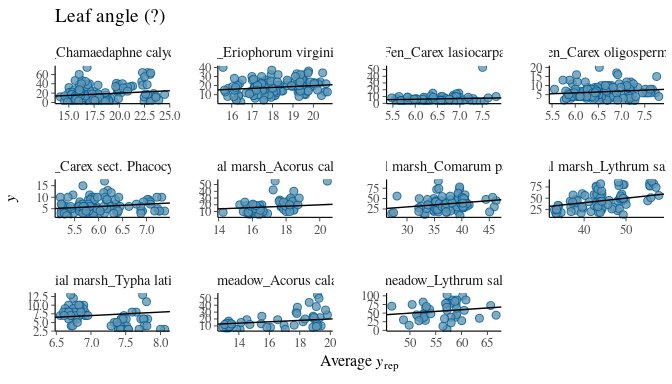


**Figure S38**: Fit between observed and predicted leaf angle values for each species in their. Line represent a 1:1 line.


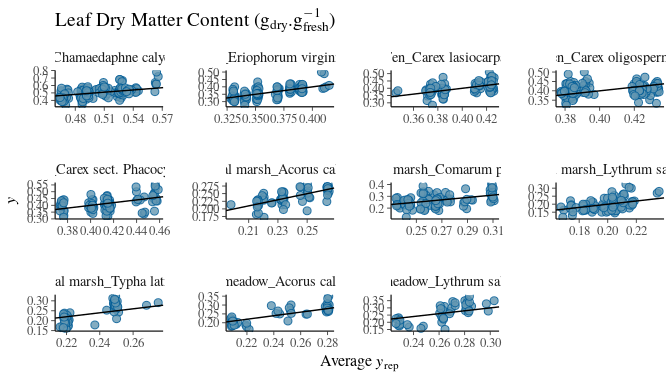


**Figure S39**: Fit between observed and predicted leaf dry matter content values for each species in their. Line represent a 1:1 line.


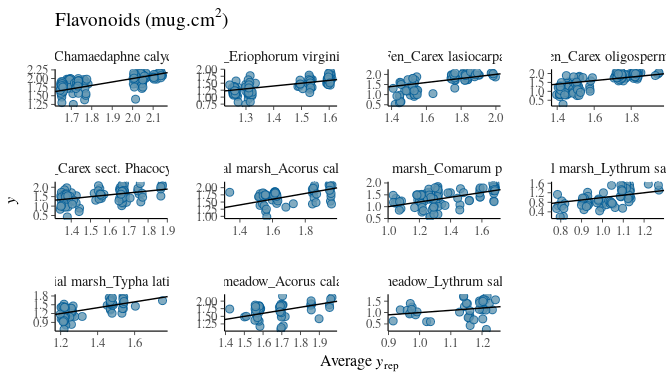


**Figure S40**: Fit between observed and predicted flavonoids values for each species in their. Line represent a 1:1 line.


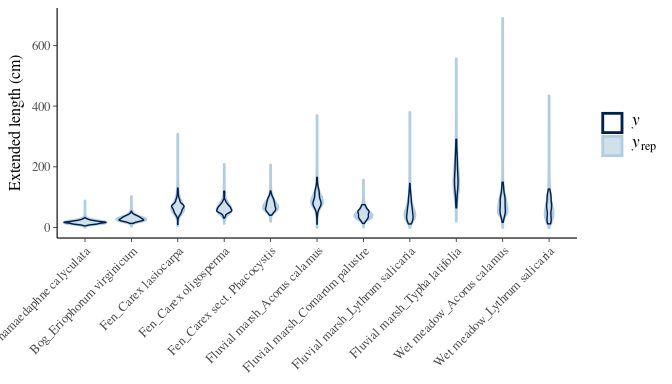


**Figure S41**: Violin plots representing the observed and predicted extended length distribution of each dominant species in their ecosystems.


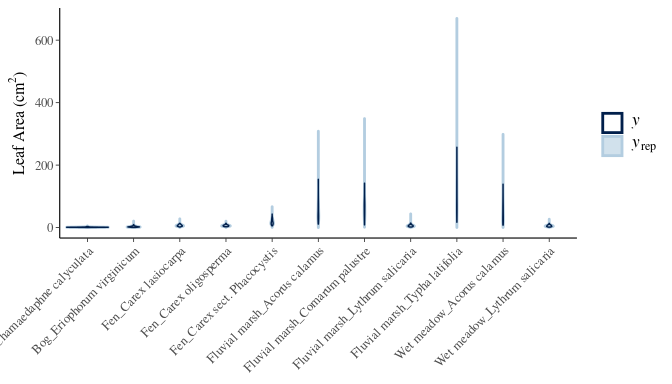


**Figure S42**: Violin plots representing the observed and predicted leaf area distribution of each dominant species in their ecosystems.


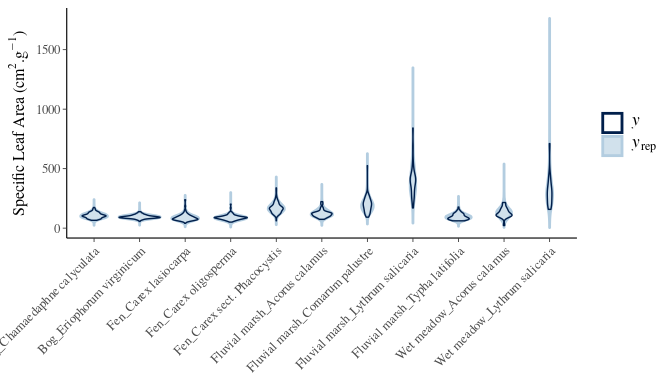


**Figure S43**: Violin plots representing the observed and predicted specific leaf area distribution of each dominant species in their ecosystems.


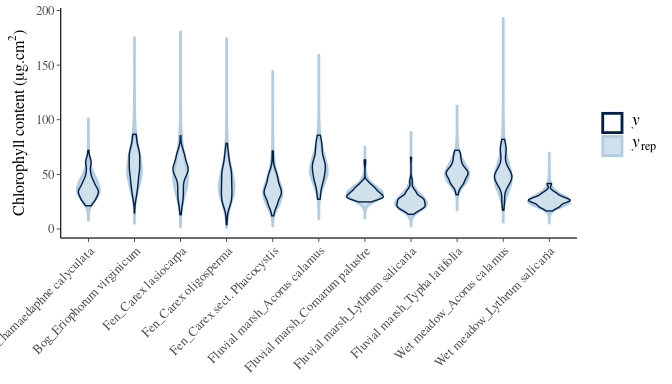


**Figure S44**: Violin plots representing the observed and predicted superficial chlorophyll distribution of each dominant species in their ecosystems.


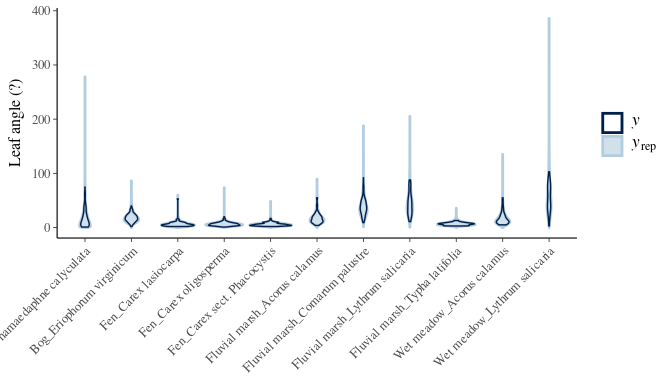


**Figure S45**: Violin plots representing the observed and predicted leaf angle distribution of each dominant species in their ecosystems.


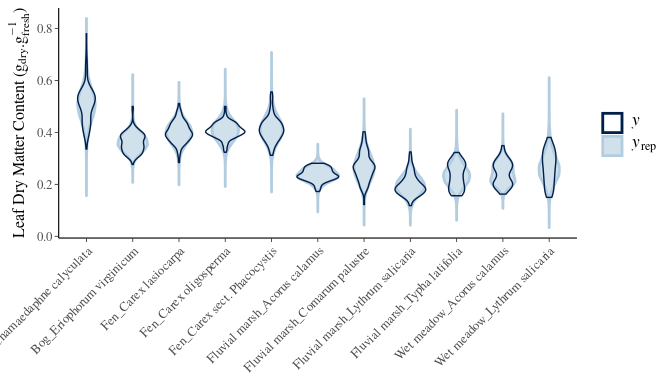


**Figure S46**: Violin plots representing the observed and predicted leaf dry matter content distribution of each dominant species in their ecosystems.


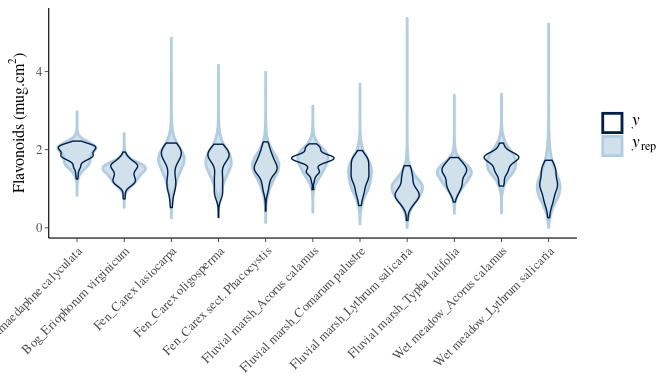


**Figure S47**: Violin plots representing the observed and predicted flavonoids distribution of each dominant species in their ecosystems.

1. Literature cited

Bürkner, P.-C. Advanced Bayesian Multilevel Modeling with the R Package brms. R J. 10, 395–411 (2018).

Gelman, A. et al. Bayesian data analysis. (Chapman and Hall/CRC, 2013).

Rigby, R. A. & Stasinopoulos, D. M. Generalized additive models for location, scale and shape. Appl. Stat. 54, 507–554 (2005).

Smyth, G. K. Generalized Linear Models with Varying Dispersion. J. R. Stat. Soc. Ser. B 51, 47–60 (1989).
